# Energy Sources and Thermal Management Technologies for Electric Vehicle Batteries: A Technical Review

**DOI:** 10.1002/gch2.202500083

**Published:** 2025-06-02

**Authors:** Md Atiqur Rahman, Gurrala Mohith Venu Reddy, Rajeshwari Chatterjee, Soumili Hait, S. M. Mozammil Hasnain, Prabhu Paramasivam, Leliso Hobicho Dabelo

**Affiliations:** ^1^ Department of Mechanical Engineering Vignan's Foundation for Science Technology and Research Vadlamudi Guntur Andhra Pradesh 522213 India; ^2^ Department of Electronics and Communication Engineering Vignan's Foundation for Science Technology and Research Hyderabad Telangana 508284 India; ^3^ Department of Chemical Engineering Birla Institute of Technology Ranchi Mesra 835215 India; ^4^ Marwadi University Research Center Department of Mechanical Engineering Faculty of Engineering and Technology Marwadi University Rajkot Gujarat 360003 India; ^5^ Department of Research and Innovation Saveetha School of Engineering SIMATS Chennai Tamil Nadu 602105 India; ^6^ Department of Mechanical Engineering Mattu University Mettu 318 Ethiopia

**Keywords:** advanced cooling technologies for fast charging, battery thermal management system, electric vehicle energy storage, lithium‐ion battery cooling, phase change materials for thermal regulation

## Abstract

Efficient thermal management of high‐power lithium‐ion batteries (LiBs) is critical for ensuring safety, longevity, and performance in electric vehicles (EVs). Battery thermal management systems (BTMS) play a crucial role in regulating temperature, as LiBs are highly sensitive to thermal fluctuations. Excessive heat generation during charging and discharging can degrade battery performance, reduce lifespan, and pose safety risks. Traditional cooling methods, such as air and liquid cooling, often require additional power and complex components, making them less effective for high‐energy–density batteries. As a result, recent advancements focus on immersion, indirect, and hybrid cooling solutions. Among these, phase change material (PCM)‐based BTMS has emerged as a promising passive cooling approach. PCMs efficiently absorb and store heat, maintaining optimal battery temperature without external power. Their thermal performance is further enhanced by integrating expanded graphite (EG) fillers, metal foams, or fins, improving heat dissipation. This review examines recent progress (2019–2024) in BTMS technologies, with a particular focus on PCM applications in fast‐charging conditions. It also discusses BTMS performance under extreme environments, such as high temperatures, sub‐zero conditions, and abuse scenarios. Future research directions are highlighted to optimize BTMS for next‐generation EVs, ensuring improved battery safety, efficiency, and thermal stability.

## Graphical abstract

Overview of Battery Thermal Management System (BTMS) methods, including air, liquid, cold plate, and hybrid cooling strategies—highlighting direct/indirect liquid techniques and hybrid integrations like PCM, refrigerant, and heat pipe systems.

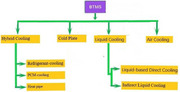

## 1 Introduction

Nations around the world are classified as either developed or developing based on their per capita energy consumption. Fossil fuels have been extensively used for land, water, air transportation, and other utilities. Based on their energy usage per capita, countries are categorized as either developed or developing. In addition to numerous other utilities, fossil fuels have been widely utilized for air, sea, and land transportation.^[^ ^1^ ^]^ While the fossil fuel‐driven economy has generated immense wealth and driven industrial progress, it has also become a major contributor to the rise in global temperatures. The combustion of these fuels releases exhaust gases that lead to catastrophic environmental changes. For decades, scientists have expressed growing concern over climate change. Current projections suggest that global temperatures could rise by 1.4–5.8 °C at the rate of acceleration by the end of the 21st century, according to reports from the IPCC. This increase in global temperatures is linked mainly to the rise in carbon dioxide levels, which unsustainable energy consumption has driven.^[^ ^2^ ^]^ The Organization for Economic Cooperation and Development also predicts global temperatures could increase by about 0.5 °C between 2030 and 2050.

The automotive sector has recently gained prominence as a vital global industry, primarily due to its significant environmental consequences. The sector's significant reliance on fossil fuels results in widespread air pollution and substantially contributes to the emission of GHGs, with CO_2_ being the most prevalent. The Environmental Protection Agency estimates that about 27% of global CO_2_ emissions are caused by transportation.^[^ ^3^ ^]^ Furthermore, 96% of global transportation still uses fossil fuels,^[^ ^4^ ^]^ underscoring the pressing need to switch to greener and more sustainable options. Since they run on electricity rather than conventional fuels, electric vehicles (EVs) have emerged as a promising alternative that can drastically reduce pollution.^[^ ^5^ ^]^

As efforts to conserve energy and reduce emissions gain momentum, the automotive industry is steadily moving away from internal combustion engines.^[^ ^6^, ^7^ ^]^ NEVs, which utilize renewable energy sources, are being introduced as replacements for vehicles that rely on fossil fuels.^[^ ^8^, ^9^ ^]^ Among the different energy storage systems, LiBs are the most favorable technology for NEVs, including HEVs and pure BEVs. Their long cycle life, high energy and power density, and eco‐friendly qualities account for this demand.^[^ ^6^, ^10^, ^11^, ^12^ ^]^

The global effort for more environmentally friendly modes of transportation that aim to lower greenhouse gas emissions and advance sustainability is strongly related to the growth of electric cars (EVs).^[^ ^13^ ^]^ There are numerous energy storage options for EVs, including flywheels, electrochemical batteries, hydrogen fuel cells, and ultracapacitors.^[^ ^14^ ^]^ LiBs are widely utilized because of their superior energy density and affordability. Regardless of these benefits, the limited driving range of EVs, typically between 200 and 350 km on a full charge, remains a significant drawback. Batteries are the most vital component of an EV and the most expensive, posing a key challenge to the further development of electric vehicles.^[^ ^15^ ^]^ Consequently, the future success of electric vehicles largely depends on developing more efficient, affordable, and powerful batteries, which would enhance vehicle range and overall performance.

In summary, the energy storage scheme is vital for EVs, requiring durability, affordability, lightweight design, safety, and efficiency. Batteries produce substantial heat during fast charging and discharging cycles due to electrochemical reactions and high current flow. Excessive heat can lead to serious risks, including overheating or potential explosions. Additionally, extreme temperatures can negatively impact battery performance, reducing the vehicle's driving range. To mitigate these challenges, a VTM is implemented to enhance safety and efficiency. This system plays a critical role in preventing overheating, thus extending the lifespan of components and ensuring overall safety.^[^ ^16^ ^]^ As a result, improving battery production sustainability is vital to guaranteeing the long‐term sustainability of electric vehicles and their capacity to reduce environmental impact.^[^ ^17^ ^]^

Multiple factors affect an EV battery pack, including efficiency, with temperature playing a crucial role. A typical lithium‐ion (Li‐ion) EV battery pack functions most efficiently between 15 and 35 °C temperature. Operating beneath 15 °C can cause a reduction in overall capacity and a surge in the battery's internal resistance.^[^ ^18^ ^]^ On the other hand, temperatures exceeding 35 °C can trigger irreversible chemical reactions within the battery pack and heighten the risk of thermal runaway.^[^ ^19^ ^]^ Moreover, excessive heat can accelerate the decline in battery capacity.^[^ ^20^ ^]^ Given the profound impact that temperature has on the performance of EV battery packs, ongoing improvements in cooling systems are essential to enhance both the lifespan and safety of the batteries.

BTMS is critical in ensuring electric vehicles' safe and efficient operation (EVs). A well‐designed BTMS fulfils several essential functions:^[^ ^21^, ^22^ ^]^

1. Temperature Control: Ensures the battery pack operates within its ideal temperature range, averting overheating throughout charging or use while minimizing the impact of cold temperatures on performance.
2. Enhanced Safety: By regulating temperatures, the BTMS lowers the risk of battery deterioration and thermal runaway—a hazardous state that can cause rapid heating and the release of flammable gases—ultimately prolonging battery life.
3. Optimized Performance: Keeping the battery at an optimal temperature allows EVs to maintain peak performance, enhancing power output and driving range.

Recent advancements in research on BTMS have led to significant progress, allowing these systems to be approximately characterized into several types: active or passive systems,^[^ ^10^ ^]^ parallel or series configurations,^[^ ^23^ ^]^ heating or cooling systems, and those utilizing air, water, or PCM.^[^ ^24^ ^]^ Additionally, hybrid strategies that integrate multiple methods^[^ ^25^ ^]^ have been developed, enhancing the effectiveness of BTMS. The design of BTMS plays a vital role in influencing costs, optimizing heat transfer efficiency, managing energy effectively, preserving battery health, and increasing energy density. As the demand for faster charging and longer journeys escalates, the importance of BTMS continues to grow.^[^ ^26^ ^]^

**Figure 1:**
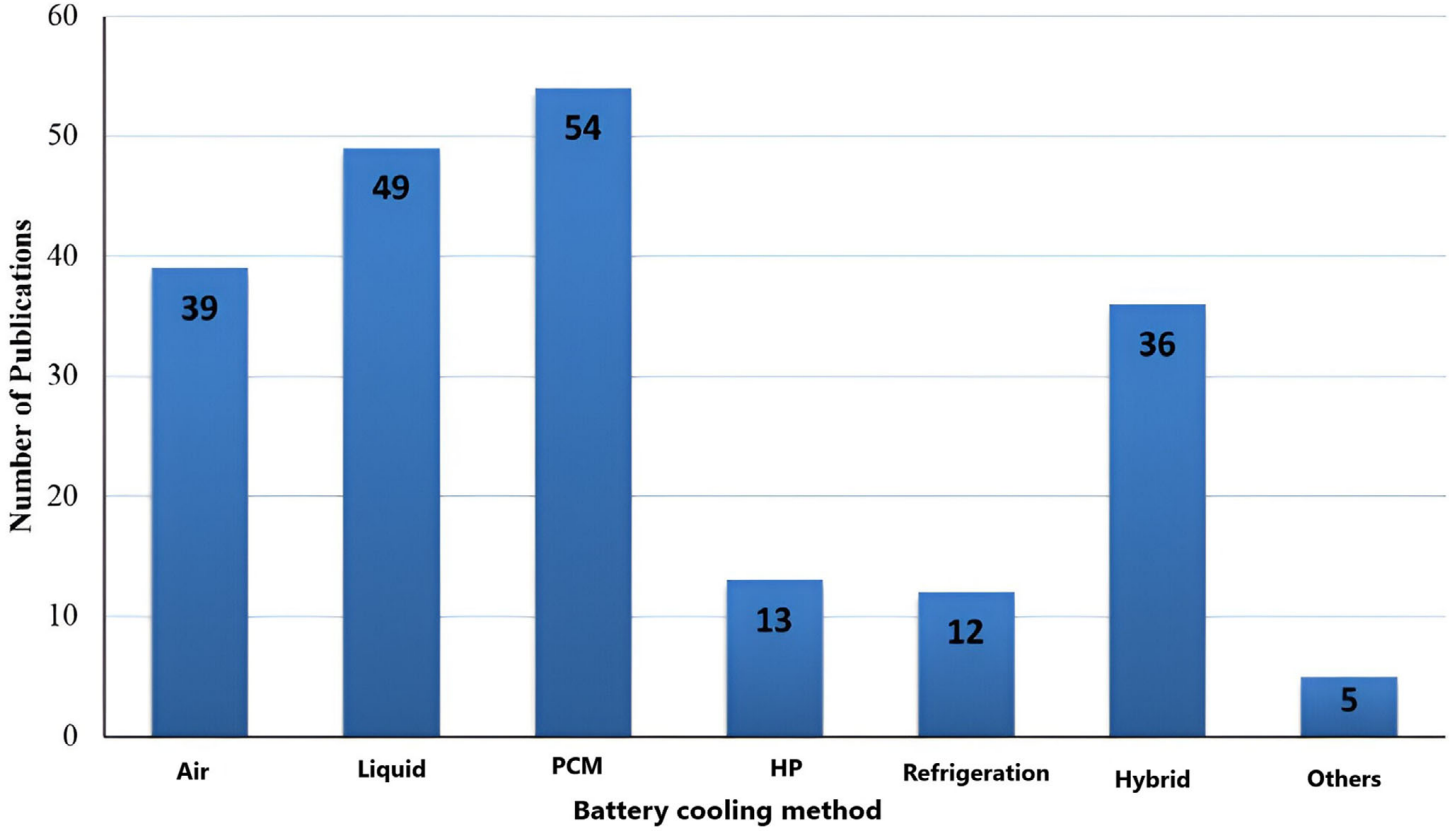
Classification of BTMS review studies. Reproduced with permission^[^ ^27^ ^]^ 2021 Elsevier Ltd.

This study recommends creating an effective BTMS for installing high‐energy–density LiBs in NEVs. The first part of the conversation looks at how temperature affects the functionality of individual batteries and battery systems. The advantages and drawbacks of the most recent developments in liquid, air, and PCM‐based cooling systems are then discussed. Statistics on the battery cooling techniques presented are shown in **Figure** **1**.^[^ ^27^ ^]^ This article also examines the integration of BTMS with VTM systems in greater facets. Last, issues and potential paths for BTMS are discussed, emphasizing avoiding thermal runaway. This paper intends to offer valuable insights into designing and optimizing BTMS for NEVs.

## 2 Lithium‐Ion Battery

This section deals with the basics of LiB, starting with the LiB's fundamental and chemical reactions causing heat generation in the LiBs with the consequences of rising temperatures. The side effects of this rising temperature are further discussed. Finally, different problems associated with the rising cell temperature are discussed. Further, the different cooling strategies of LiB, such as air, water, and hybrid cooling, have been discussed. Last, recent trends in estimating state‐of‐charge (SOC) have been discussed, including OCV readings, Coulomb counting, extended kalman filters (EKFs), and various approaches using artificial intelligence algorithms.^[^ ^28^ ^]^

### 2.1 Definition

**Figure 2:**
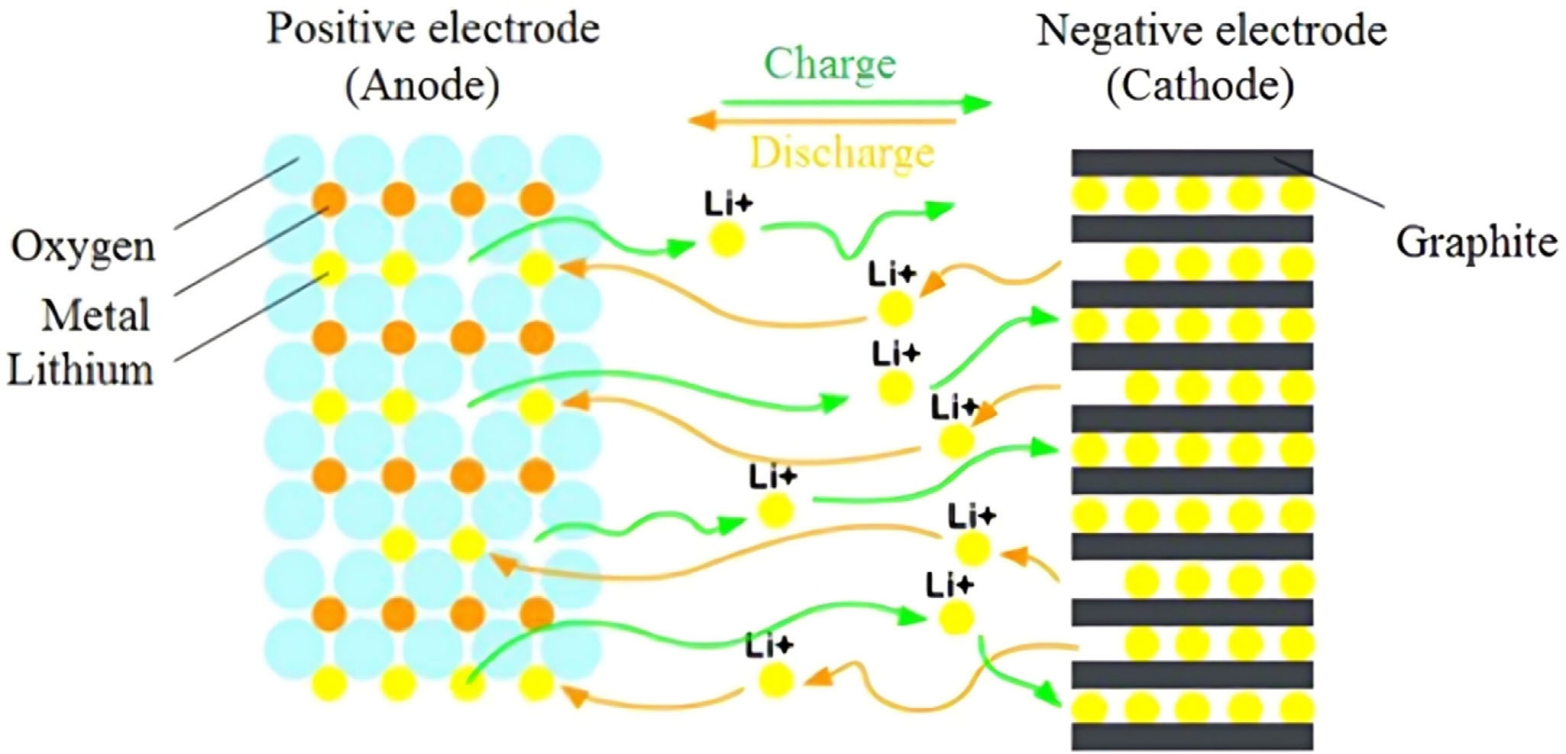
Schematic of a LiB. Reproduced with permission^[^ ^29^ ^]^ 2016 Elsevier Ltd.

LiB is an electrochemical device that comprises four essential components: a negative electrode (anode), a positive electrode (cathode), a separator, and an electrolyte (**Figure** **2**).^[^ ^29^ ^]^ The primary factors influencing this process include ion transport through three key areas: 1) within the solid electrodes, 2) at the electrode/electrolyte boundary on both the anode and cathode, and 3) through the electrolyte, involving the solvation and desolvation of Li^+^ ions.^[^ ^30^ ^]^ During discharging, the anode releases electrons to the external circuit while the cathode obtains electrons from the circuit. The separator prevents the direct flow of electrons between the electrodes while minimally interfering with other processes. Furthermore, a thin passivation layer called the solid electrolyte interphase (SEI) forms on the carbon anode during the initial charge. This layer slows the reaction rate, reduces current flow, and gradually thickens as the battery ages.^[^ ^31^, ^32^ ^]^

### 2.2 Temperature‐Induced Degradation

Temperature affects several LiB deterioration processes. Arrhenius equations^[^ ^33^, ^34^ ^]^ or empirical equations that fit experimental data^[^ ^31^, ^35^, ^36^, ^37^, ^38^ ^]^ are frequently used to characterize these temperature effects. At higher temperatures, the anode's SEI layer expands rapidly, becoming increasingly porous and unstable. Conversely, lower temperatures hinder ion diffusion and intercalation, elevating the risk of lithium plating and dendritic lithium growth. On the cathode side, high temperatures during cycling lead to phase transitions, metal dissolution, binder decomposition, and the creation of the cathode electrolyte interphase. The electrolyte, commonly composed of ethylene carbonate (EC) and dimethyl carbonate (DMC) in equal proportions with 1 m LiPF_6_, is prone to decomposition at high temperatures, producing gaseous byproducts such as CO_2_. In general, elevated temperatures accelerate most degradation processes. While lowering the temperature can mitigate degradation by slowing the diffusion of active species and modifying reaction dynamics, it may also intensify deprivation if metallic lithium accumulates on the anode. Additionally, because of slower reaction kinetics and increased transport limits, working at lower temperatures results in higher overpotentials, which raise heat generation and reduce energy efficiency.^[^ ^39^ ^]^

The development of the SEI at the interface between the anode and electrolyte is a key factor contributing to lithium‐ion battery degradation across various cycling conditions. This protective layer increases the cell's impedance, leading to a reduction in power output. The lithium consumed during SEI formation is irreversible, contributing to gradual capacity loss. The temperature has a major influence on the electrochemical reduction of the electrolyte, leading to the development of a complex blend of lithium compounds such as LiF, Li₂CO₃, and (CH₂OCO₂Li)₂.^[^ ^40^ ^]^ Initially, the SEI layer serves as a protective barrier, but with repeated cycling, it becomes unstable, thickens, and may obstruct the electrode's pores or even penetrate the separator. This increases impedance due to reduced active surface area and potential safety risks. When exposed to high temperatures (60 °C and above), the disbanding and breakdown of SEI components, including Li₂CO₃, can weaken the protective layer, fragmenting into distinct regions.^[^ ^41^ ^]^

The thermal breakdown of the carbonate‐based electrolyte yields several byproducts, including CO_2_, CO, C_2_H_4_, hydrogen, alkylfluorides, and H_3_PO_3_F, in addition to the SEI that forms on the graphite anode. Gachot et al.^[^ ^42^ ^]^ recovered lithium methyl carbonate from the separators of Li/chromium‐based oxide (Li/CBO) cells that had undergone cycling at 55 °C. LiPF_6_ becomes unstable at temperatures above 60 °C, leading to its dissociation into PF5, which can break down the protective SEI layer and expose fresh graphite surfaces.^[^ ^43^ ^]^ EC/DMC/diethyl carbonate mixtures' NMR spectra of 1.0 m LiPF_6_ demonstrate that these thermally induced breakdown processes intensify at 85 °C.^[^ ^44^ ^]^ In addition to impairing battery efficiency, producing gaseous byproducts during SEI formation and electrolyte degradation also degrades the anode and cathode's mechanical qualities.

For a battery subjected to high temperatures for prolonged periods of time, electrode delamination and particle cracking become major issue when parasitic reaction byproducts build up. Delamination was noted by Pieczonka et al. in a LiNi_0.5_Mn_1.5_O_4_ cathode bound with polyvinylidene fluoride, where the cathode separated from the aluminum current collector after being stored in the electrolyte at 60 °C for 3 weeks and during cycling.^[^ ^45^ ^]^ Rapid charging/discharging cycles, temperature swings, and volume variations in the electrode material cause stress, eventually resulting in cracks and delamination when the binders cannot keep the electrode together.^[^ ^46^ ^]^ Carbon additives in the cathode are electrochemically reactive with PF6‐ions, resulting in structural changes to the cathode. These effects are more pronounced at 45 °C than 25 °C.^[^ ^47^ ^]^ Other elements contributing to structural instability, such as the interface between electrolyte oxidation products and corrosion induced by hydrofluoric acid (HF), tend to be expedited at elevated temperatures.

Thermal runaway can happen when the temperature reaches a critical threshold. An uncontrolled positive feedback loop is created when the pace of exothermic reactions and the ensuing temperature increase contribute to thermal runaway. Thermal runaway is characterized by electrolyte breakdown, anode/electrolyte and cathode/electrolyte reactions, interactions with the binder, and decomposition of the SEI layer.^[^ ^48^ ^]^ The breakdown of the SEI layer, which can be caused by physical damage, overcharging, or overheating, is linked with the exothermic breakdown of unstable constituents such (CH_2_OCO_2_Li)_2_, which is trailed by more exothermic interactions between the anode and electrolyte.^[^ ^49^ ^]^ The degradation of the SEI layer, which may result from physical damage, overcharging, or excessive heat, is linked to the exothermic decomposition of unstable compounds like (CH_2_OCO_2_Li)_2_, followed by further exothermic reactions between the anode and electrolyte.^[^ ^49^ ^]^ While most polymer separators dissolve at temperatures above 130 °C, some are specifically engineered to preserve their structural integrity and avert short circuits. For instance, trilayer polypropylene–polyethylene–polypropylene (PP‐PE‐PP) separators can be designed to deactivate once the temperature surpasses a definite threshold. The electrochemical activity of the cell is essentially stopped when the polyethylene (PE) layer melts because the separator loses its ionic *k*_p_ and becomes nonporous.

Meanwhile, the polypropylene (PP) layers retain their mechanical properties due to their higher melting point, averting separator shrinkage and short‐circuit formation.^[^ ^50^ ^]^ An alternative method for inducing thermal shutdown involves placing an additional material layer amongst the cathode and the current collector. A material with good electrical *k* at moderate temperatures that consists of spiky nickel nanoparticles covered in graphene in a polymer matrix was suggested by Chen et al.^[^ ^51^ ^]^ At high temperatures (such as 70 °C in their study, though this can vary depending on the polymer matrix and the nickel‐to‐polymer ratio), the electrical *k* of the material decreases drastically by seven to eight orders of magnitude within just 1 s, successfully turning off the cell and averting thermal runaway. If thermal runaway is not clogged before the separator melts, the cell experiences an internal short circuit that damages the cathode. Electrolyte decomposition becomes severe between 250 and 350 °C during thermal runaway, rapidly producing gaseous byproducts. This increases internal pressure, releasing flammable vapors into the surrounding environment.^[^ ^52^ ^]^

### 2.3 Thermal Issues of LiBs

**Figure 3:**
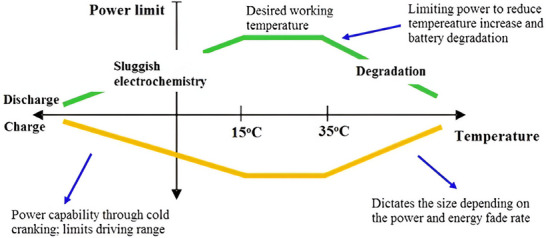
Battery operating range. Reproduced with permission^[^ ^29^ ^]^ 2016 Elsevier Ltd.

The performance of LiBs is heavily influenced by operating temperature and voltage. As depicted in **Figure** **3**,^[^ ^29^ ^]^ LiBs operate optimally within a specific temperature range. Outside of this range, irreversible damage can occur, potentially leading to thermal runaway.

Temperature significantly influences the chemical processes happening inside a cell.^[^ ^29^ ^]^ When the temperature rises, these reactions speed up, leading to greater capacity and power output. However, this also leads to more heat being released, which in turn raises the temperature even further.^[^ ^53^ ^]^ If heat is not expelled faster than it is generated, the temperature will rise, inevitably leading to thermal runaway.^[^ ^54^ ^]^ Safety is a critical concern in modern BEVs.^[^ ^55^ ^]^

**Figure 4:**
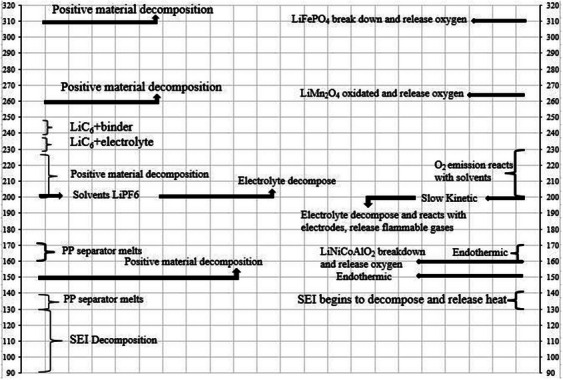
Exothermic reactions and thermal stability of LiB.

**Figure** **4** illustrates how exothermic reactions in LiB vary with temperature when heat is not effectively managed.^[^ ^29^ ^]^ Initially, SEI decomposes, followed by reactions between the electrode and electrolyte, which produce combustible gases. At 130 °C, the separator melts, causing a short circuit between the electrodes. Low temperatures decrease the battery's available energy in cold environments and surge its internal resistance.^[^ ^56^ ^]^ Figure 4 depicts battery performance declines significantly as the operating temperature drops. Jaguemont et al.^[^ ^56^, ^57^ ^]^ note that extremely low temperatures can freeze the battery, reducing its capacity, while very high temperatures can harm the active chemical apparatuses of the battery. Additionally, the heat generated in battery cells and packs, especially in high‐current uses, necessitates effective cooling.^[^ ^29^ ^]^

Jaguemont et al.^[^ ^49^ ^]^ observed that extremely cold conditions can freeze the battery, which reduces its capacity. On the other hand, very high temperatures can harm the battery's chemical components. Furthermore, they emphasize that the need for battery cooling is determined mainly by the heat produced within the cells and packs, especially during high‐current use. Battery's performance can vary significantly with temperature. As observed, the battery's capacity drops sharply as the operating temperature decreases. Careful temperature control is essential, as excessive and insufficient heat can cause significant issues.^[^ ^26^, ^58^, ^59^ ^]^

### 2.4 Generated Heat Loss in LiB

During the charging and discharging cycles, LiBs yield significant heat due to internal electrochemical processes.^[^ ^58^, ^59^ ^]^ The battery's temperature rises significantly as a result of this heat generation. The C‐rate governs the rates at which current is added/removed from the battery throughout these cycles. A higher C‐rate corresponds to a larger current, resulting in a faster heat buildup. Studies have shown that for a 10 Ah LiB, the heat generated at charging rates of 3C, 5C, and 8C is ≈10.5, 25, and 54 W, respectively.^[^ ^60^ ^]^ The heat generated during charging and discharging (*Q*_gen_) is determined by the combined effect of reversible heat (*Q*_rev_) and irreversible heat (*Q*_irr_). Reversible heat is caused by entropy generation, while irreversible heat results from Joule heating, as expressed below in Equation (1):^[^ ^61^ ^]^

(1) $$Q_{\mathrm{gen}}=Q_{\mathrm{irr}}+Q_{\mathrm{rev}}=I\left(U_{\mathrm{oc}}-V_{\mathrm{bat}}\right)-IT\frac{\partial U_{\mathrm{oc}}}{\partial T}$$

𝐼 = current flowing through the battery during charging or discharging (*I > 0*)

𝑈_𝑜𝑐_ = open‐circuit voltage, 𝑉_𝑏𝑎𝑡_ = voltage across the battery.

𝑇 = temperature of the battery,

𝜕𝑈_𝑜𝑐_/𝜕𝑇 = entropic coefficient (open‐circuit voltage changes with respect to temperature).

The disparity between *U* and *V*_bat_ signifies the overall overpotential of a battery, which arises from processes such as charge transfer reactions at the electrode/electrolyte interfaces,^[^ ^43^ ^]^ the movement and migration of Li^+^ ions through the electrolyte,^[^ ^62^ ^]^ as well as their diffusion and migration within the electrodes,^[^ ^63^ ^]^ along with Ohmic losses.^[^ ^64^ ^]^ A significant factor contributing to irreversible heat generation is resistive (Joule) heating Equation (2):^[^ ^65^ ^]^

(2) $$Q_{joule}=I^{2}R$$

where *R* represents the cell resistance, since fast charging involves higher charging currents, it leads to increased heat generation due to the quadratic relationship between the rate of irreversible heat generation and the current. The heat produced or absorbed during the reversible process, known as entropic heat, results from the reversible entropy change (Δ*S*) that occurs during electrochemical reactions.^[^ ^65^ ^]^ Once the Δ*S* is determined,^[^ ^49^ ^]^ the reversible heat is calculated from Equation (3):^[^ ^66^ ^]^

(3) $$Q_{rev}=I\frac{T\Delta S}{nF}$$

*T* = absolute temperature,

*n* = number of electrons tangled in the electrochemical reaction,

*F* = Faraday's constant.

To evaluate thermal performance based on the temperature evolution during a fixed 60‐min cycle for each discharge rate (heat generation rate), a performance enhancement factor *θ* was defined, as shown in Equation (4):^[^ ^67^ ^]^

(4) $$\theta \left(t\right)=\frac{T_{cell\left(t\right)}-T_{\mathrm{ref}\left(t\right)}}{T_{\mathrm{initial}\left(t\right)}-T_{\mathrm{ref}\left(t\right)}}$$

where *T*_cell_ is the temperature at *t* = 60 min, *T*_initial_ is the initial temperature of the cell at *t* = 0 min, and *T*_ref_ is the reference temperature, which was the average ambient temperature kept constant.

Nazari et al.^[^ ^68^ ^]^ engaged a mathematical model to analyze heat generation in LiB with graphite anodes, including lithium iron phosphate, lithium manganese oxide, and lithium cobalt oxide chemistries. Their research revealed that total heat generation across all tested cells remained within a similar range. At low C‐rates, reversible heat generation was predominant, whereas, at high C‐rates, irreversible heat became the main contributor. Since the lifespan of LiB is highly dependent on cell temperature, the US‐NREL developed ageing models^[^ ^69^ ^]^ to assess the effects of charge/discharge cycles and temperature on battery longevity. The research demonstrated that lowering the average temperature from 35 to 20 °C during storage or operation can almost double the battery's lifespan.

## 3 Battery Thermal Management

As previously mentioned, a BEV's battery system requires a regulated environment where temperature fluctuations are carefully managed, preventing thermal runaway to enhance safety, durability, and efficiency.^[^ ^70^ ^]^ The batteries are designed with standardized shapes and dimensions to gain widespread acceptance in the industry.^[^ ^29^ ^]^ In some applications, battery systems operate under harsh conditions, such as rapid charging/discharging and extreme temperatures, which can increase the risk of system failure. A BTMS is essential to regulate the battery's temperature in an optimum range. The choice of heat transfer medium plays a vital role in deciding the performance and cost‐efficiency of the BTMS. According to foundational research, multiple cooling methods are available for the battery system. The subsequent sections will thoroughly overview conventional thermal management techniques and evaluate their heat dissipation capabilities.

Prismatic cells seem the best option for cooling automobiles because of their comparatively large surface area, making transferring heat from the cell's interior to the outside easier. Prismatic cells are considered the most suitable choice for cooling vehicles due to their larger surface area, which enhances heat dissipation from the cell's interior to its exterior. Cylindrical cells are commonly used primarily due to their cost‐effectiveness, durability, safety, availability, and advanced production techniques. Companies like Tesla and BMW Mini notably rely on these cells. In vehicle applications, cells are organized in various configurations and integrated with safety and control mechanisms to construct a battery module. These modules are assembled with added control electronics, a thermal regulation system, and power components to develop a complete battery pack.

The basic types of BTMS are listed below.

### 3.1 Air Cooling

Air‐cooling methods are commonly used in BTMS for NEVs because of their simple structure, lightweight characteristics, and comfort of upkeeping. These features contribute to their affordability and reliability.^[^ ^71^ ^]^ Air‐cooling techniques are classified into two types: forced convection and natural convection.^[^ ^26^ ^]^ In natural convection, air naturally circulates the battery pack to cool it.^[^ ^72^ ^]^ Nonetheless, research has indicated that natural convection does not effectively dissipate BTMS of NEVs.^[^ ^29^ ^]^ In contrast, forced convection offers superior heat exchange efficiency, as it utilizes air pumps and works in tandem with an evaporator to circulate cooling or heating air more effectively.^[^ ^72^ ^]^ Due to its enhanced performance in BTMS, forced convection is widely used in the automotive industry. Forced convection can be further classified into passive and active air convection.^[^ ^29^ ^]^

The outdoor temperature impacts the passive system's performance, frequently drawing air from the vehicle's cabin or the surrounding environment.^[^ ^73^ ^]^ On the other hand, the active configuration utilizes pre‐conditioned air sourced from either a heater or the HVAC system's evaporator. More efficient thermal regulation is provided by this pre‐treated air, particularly in harsh environments like below‐freezing or above‐45 °C temperatures. The following part will go over the specifics of this system.

**Figure 5:**
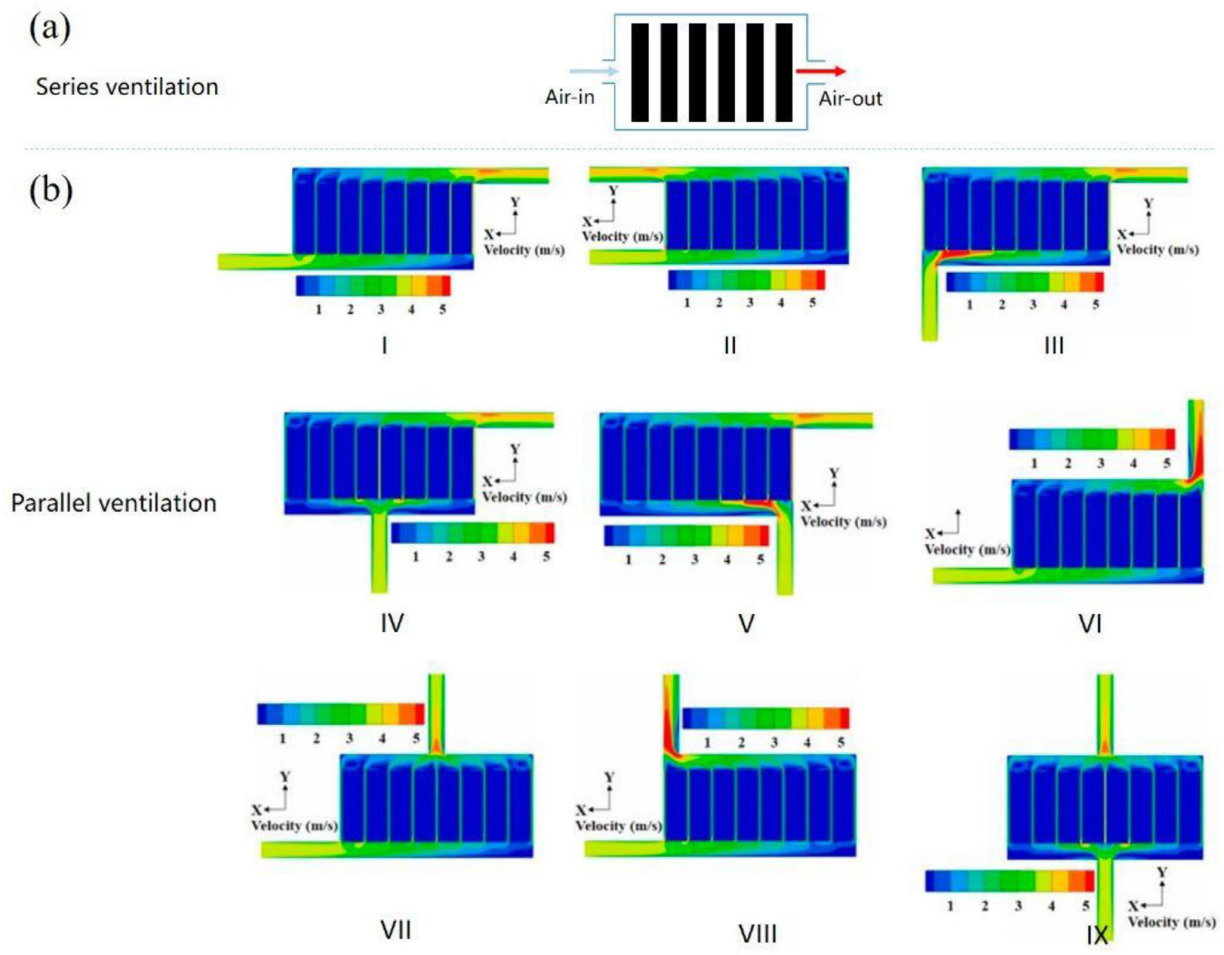
The conventional air‐cooling arrangement of BTMs. a) Ventilation in series;^[^ ^74^ ^]^ b) Ventilation parallel.^[^ ^23^ ^]^

Moreover, the air duct design plays a vital role in the effectiveness of the BTMS. In NEVs, air duct configurations are generally categorized into parallel and series ventilation, as illustrated in **Figure** **5**a,b.^[^ ^23^ ^]^ Pesaran et al. analyzed the thermal performance of battery packs with these two duct arrangements.^[^ ^74^ ^]^ Their findings suggest that a BTMS utilizing parallel ventilation achieves lower peak temperatures and a more balanced temperature dispersal than series ventilation systems. Considering the cylindrical battery's geometric dimensions, a new axial flow air cooling system was proposed.^[^ ^75^ ^]^ Studies have shown that axial ventilation optimizes space utilization within the battery pack and enhances the power density of NEVs using cylindrical batteries.^[^ ^75^, ^76^ ^]^

For the battery system to function at its best, optimizing the air‐cooling BTMS is crucial.^[^ ^77^ ^]^ Currently, advancements in computer numerical simulation technology and experimental testing aid in optimizing air‐cooling BTMS. This optimization focuses on factors such as battery placement, airflow velocities, flow pathways, and the spatial arrangement of batteries within the pack.^[^ ^26^, ^55^ ^]^

The cooling can be further done in natural and forced convection.

The parallel air‐cooled BTMS is generally categorized into nine types.^[^ ^23^, ^37^ ^]^ Several studies have examined the cooling performance of these systems using numerical techniques like CFD simulations. The velocity contours for these nine parallel air ducts are shown in Figure 8b, illustrating the significant impact of the air inlet and outlet positioning on the flow pattern.^[^ ^23^ ^]^ For BTMS III, the variation in airflow velocity within the cooling channels is most noticeable, leading to the highest temperature variations. On the other hand, BTMS VII and BTMS IX show smaller differences in airflow velocity, resulting in more consistent temperatures.^[^ ^23^ ^]^ Chen et al.’s simulation revealed that compared to the Z‐type BTMS (BTMS I), BTMS IX achieves a 4.3 °C reduction in maximum temperature and a 6.0 °C decrease in temperature variation.^[^ ^78^ ^]^

Through experimentation and optimization of factors like air intake angle, air exit angle, and battery spacing, Xie et al. improved the heat dissipation performance of the U‐type BTMS (air‐cooling).^[^ ^79^ ^]^ They found that identical spacing between the channels, a 2.5° intake angle, and a 2.5° output angle provide the best cooling performance.^[^ ^79^ ^]^ Meanwhile, Zhao et al. investigated how different battery modules in series ventilation arrangements were cooled by ventilation type, gap size, ambient circumstances, and incoming air temperature.^[^ ^79^ ^]^

The assembly of the axial air‐forced flow system is illustrated in Figure 8c.^[^ ^75^, ^80^ ^]^ Yang et al.^[^ ^75^ ^]^ created a 3D heat and mass transfer model derived from a pseudo‐2D electrochemical reaction model, concentrating on axial air‐forced convection. They found that increasing the radial gap between cylindrical batteries raised the average temperature but improved temperature uniformity. A higher airflow rate also minimized temperature disparities within the battery pack. Lu et al. examined how axial air‐forced convection improved temperature uniformity and reduced hotspots by adjusting flow paths and airflow rates. Their research revealed that enlarging the cooling channels decreased the maximum temperature, with a system comprising 59 vents proving more effective in reducing both maximum temperature and temperature variation.^[^ ^76^ ^]^

Fins are occasionally added to battery surfaces to enhance air‐cooling systems' thermal efficiency. In contrast to liquid‐cooling systems of the same volume, Chen's research indicates that adding fins can result in a 39% increase in battery weight.^[^ ^81^ ^]^ By contrast, the additional weight added by direct and indirect liquid cooling (IDC) techniques is only ≈2.95% and 7.16%, respectively.^[^ ^81^ ^]^ Fins add weight, negating the advantages of classical air‐cooling, which is frequently thought of as the simplest and lightest solution. Additionally, the use of fin‐cooling in the BTMS of NEVs is limited because of the lower energy density of battery systems.

### 3.2 Liquid Cooling

Liquid media excel in managing the temperature distribution of battery modules matched to air due to their higher thermal *k* and greater heat capacity, making them more efficient in meeting the cooling needs of large‐scale cells during high‐rate charging and discharging.^[^ ^82^ ^]^ Nonetheless, the added complexity of liquid cooling systems increases manufacturing costs. Typically, liquid‐cooling methods are categorized into direct and indirect cooling strategies.

The heat transfer effectiveness between the coolant and the battery hangs on the coolant's *k*, flow rate, density, and viscosity. Water and oil are the most commonly used coolants in liquid cooling systems. However, in recent years, nanofluids, liquid metals, and boiling liquids have shown superior performance in battery pack cooling systems, attracting significant attention. **Table** **1** represents the thermal physical characteristics of several coolants for comparison.^[^ ^83^, ^84^, ^85^ ^]^

**Table 1:** Comparison of thermal physical properties of different coolants.^[^ ^83^, ^84^, ^85^ ^]^

Property	Novec 7000	Silicone oil	Air	Water/Glycol	Mineral oil
Specific heat capacity (J kg^−1^ K^−1^)	1300	1370	1006	3323	1900
Thermal *k* (W m^−1^ K^−1^)	0.075	0.15	0.0242	0.3892	0.13
Density (kg m^−3^)	1400	920	1.225	1069	924.1
Kinematic viscosity (m^2^ s^−1^)	3.2 × 10^−7^	–	1.46 × 10^−5^	2.58 × 10^−6^	5.60 × 10^−5^

#### 3.2.1 Liquid‐Based Direct Cooling

**Figure 6:**
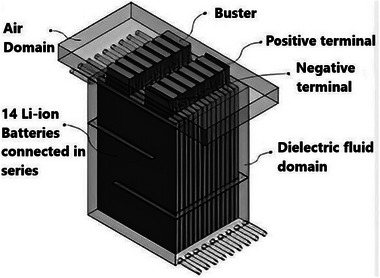
The liquid ICS (forced flow).^[^ ^87^ ^]^

Transformers have successfully employed direct cooling, sometimes liquid ICS.^[^ ^86^, ^87^ ^]^ The use of this cooling technique in electrical gadgets and NEVs has drawn a lot of interest lately. The battery module is either partly or fully deep in a cooling medium, as illustrated in **Figure** **6**, which helps improve temperature uniformity by absorbing the heat generated by the battery.^[^ ^88^ ^]^ Direct cooling also reduces complexity and simplifies system design.

To ensure efficient heat transfer, the cooling medium employed in direct cooling should possess outstanding chemical and physical characteristics, including high thermal *kp*, low viscosity, and significant heat capacity.^[^ ^89^ ^]^ It must also be electrically insulated to prevent ESC risk for the submerged battery. Moreover, environmental and safety factors require that the medium be non‐toxic, chemically stable, and non‐flammable. While water/ethylene glycol‐based coolants are commonly used in indirect cooling systems, the conductive nature of water limits its use in ICS.^[^ ^90^ ^]^ Common fluids for ICS consist of hydrocarbon, silicone, and fluorinated hydrocarbons.^[^ ^88^ ^]^

**Figure 7:**
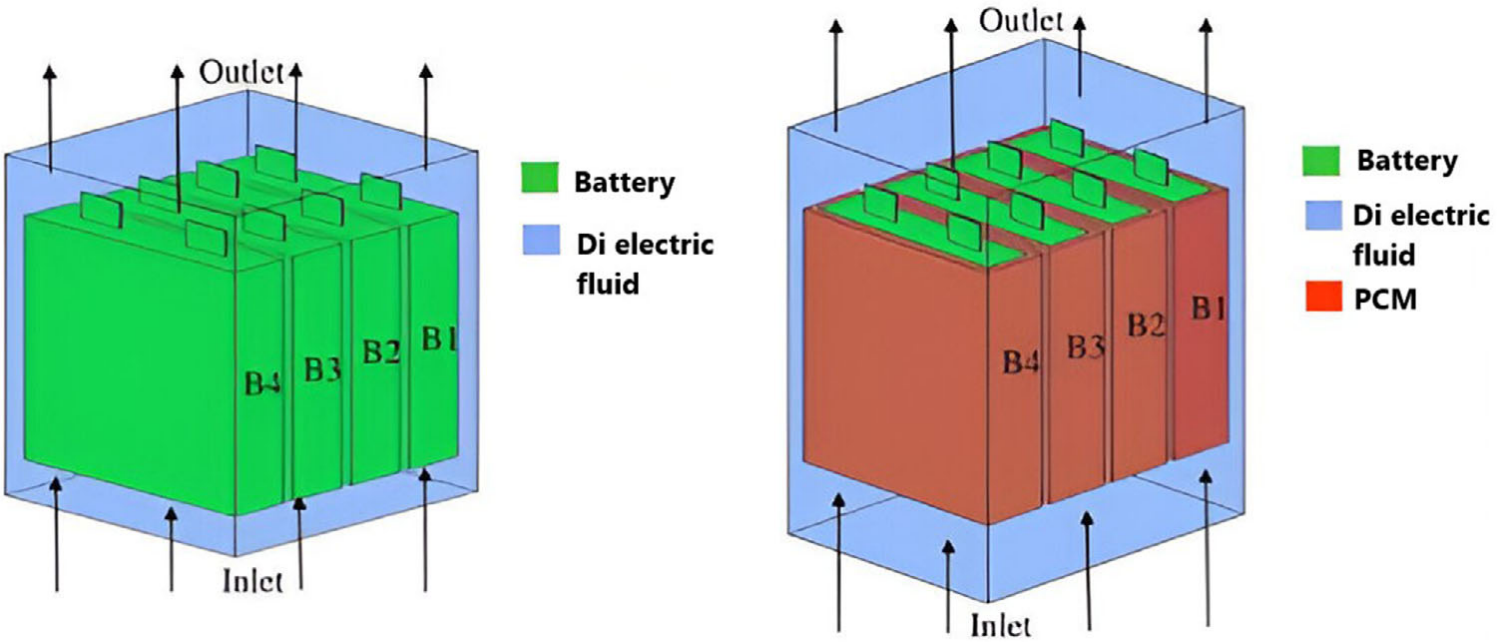
Representation of dielectric fluid immersion cooling and hybrid cooling.^[^ ^91^ ^]^

Amalesh et al. evaluated the cooling capacities of a prismatic battery module during 8C fast charging by using a hybrid cooling approach that united ICS and PCM cooling (RT35) with dielectric fluid ICS (STO‐50). As shown in **Figure** **7**, their results demonstrated that dielectric fluid ICS (STO‐50) effectively reduced the battery temperature to below 40 °C throughout 8C fast charging when the dielectric fluid was directed along the battery's length at a rate of 2 LPM, rather than across it. For optimal cooling utilizing the hybrid method for fast charging settings, the PCM's *kp* = 1 or higher. The article also indicated that raising the PCM's latent heat between 160 and 200 kJ kg^−1^ did not significantly affect the battery's cooling efficiency.^[^ ^91^ ^]^

Tan et al. managed the thermal conditions of the battery pack during fast charging by using hydrofluoroether coolant (HFE‐6120) in an ICS configuration. Their findings revealed that energy density increased by 20.3% and maximum power consumption decreased by 95.3% compared to the baseline channel. Additionally, the *T*_max_ and Δ*T* improved by 18.1% and 25.0%, respectively, using a multi‐layer structure under cross‐flow configuration.^[^ ^92^ ^]^

**Figure 8:**
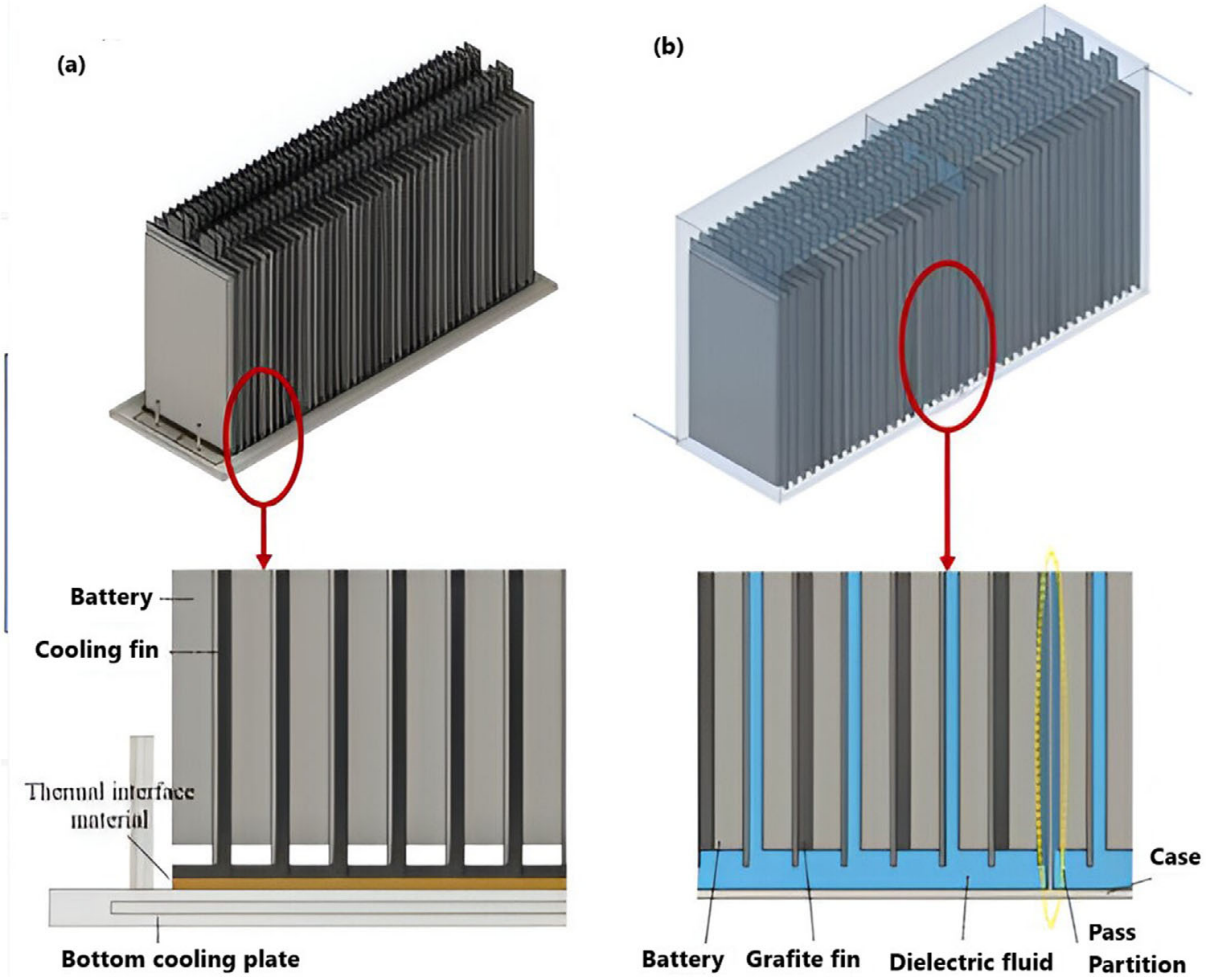
The modelling of (a) orthodox bottom cooling plate and (b) hybrid immersion cooling configuration.^[^ ^93^ ^]^

Choi et al. anticipated a hybrid ICS method that incorporates graphite fins and a pass partition, as shown in **Figure** **8**, to augment the cooling efficiency of LiB modules under demanding conditions and during rapid charging. Their analysis indicated that a bottom‐to‐top dielectric fluid flow provided more uniform fluid distribution and better heat dissipation compared to a top‐to‐bottom flow. This cooling approach significantly improved the battery pack's thermal performance, with *T*_max_ and Δ*T* remaining at 35 and 6 °C, respectively—6.7 and 3.0 °C lower than traditional cooling techniques at a 3C charging rate. Furthermore, the hybrid ICS outdid conventional methods in hydraulic performance, reducing power consumption by 61.0% and pressure by 45.4%.^[^ ^93^ ^]^

**Figure 9:**
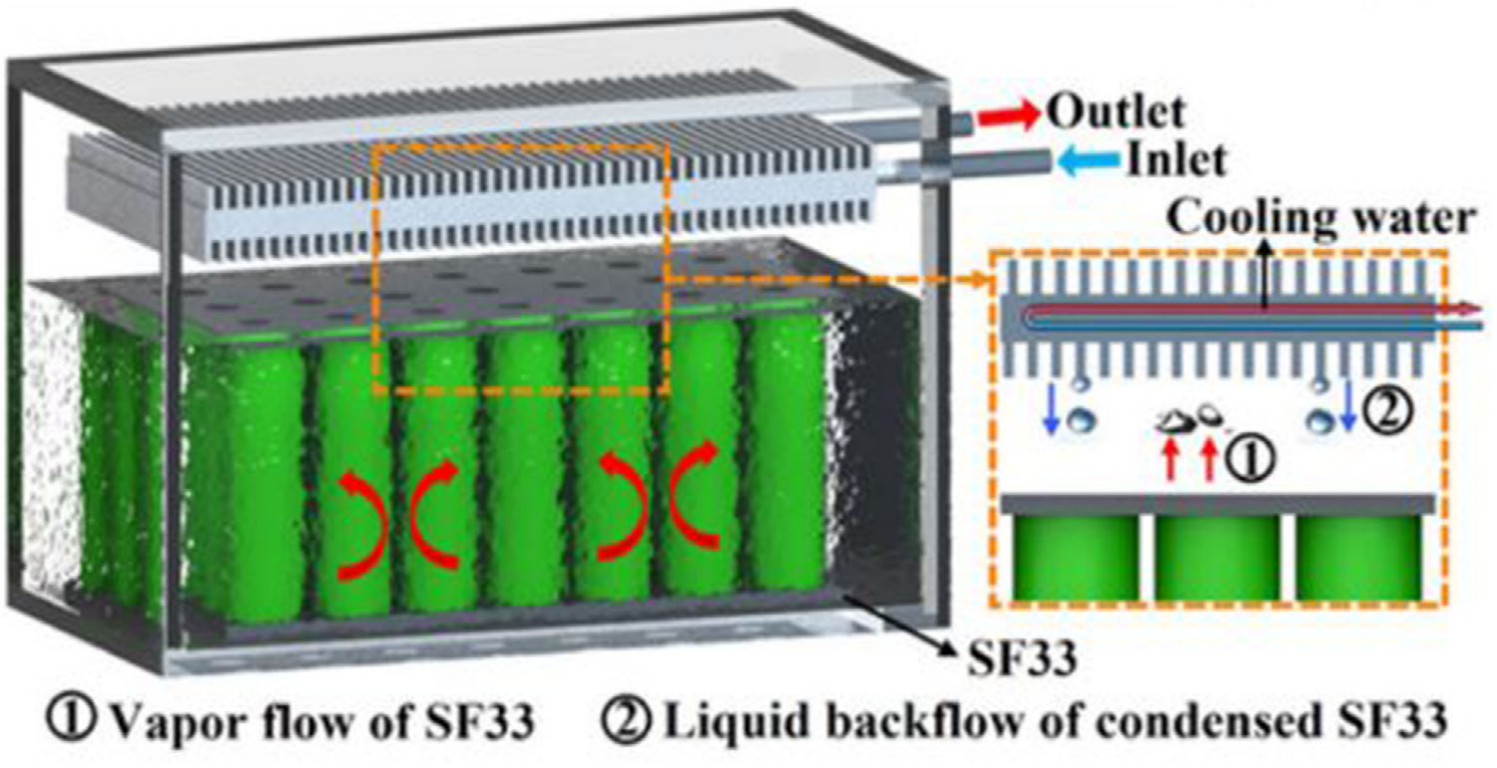
The liquid‐immersion–cooled module.^[^ ^94^ ^]^

Li et al. experimented with an ICS using FS49 fluid for fast charging a battery module (**Figure** **9**). Their findings indicated that ICS decreased the battery module's *T*_max_ by 19.6 °C at a 3C charging rate and 7.7 °C at a 2C charging rate. Additionally, comparing FAC with the novel cooling method reduced energy consumption by 14.41% and 40.37% at the same charging rates. Moreover, with a temperature variation of just 1.2 °C, the ICS demonstrated a significant advantage in maintaining a consistent temperature across the battery module.^[^ ^94^ ^]^ Ezeiza et al. introduced a novel direct cooling technique for battery modules throughout fast charging. Unlike conventional systems that immerse the battery in a cooling liquid, this method targets directly cooling the battery's external surface. Their study showed that during semi‐fast charging and discharging tests at 2C and 3C rates, the *T*_max_ and Δ*T* of the cell stayed within the optimal operating range of 38 and 1.3 °C, respectively.^[^ ^95^ ^]^

Li et al. introduced an innovative ICS using SF33 fluid. Their study showed that this ICS technique decreases the *T*_max_ and *∆T* by 19.1 and 9.8 °C, respectively, equated to FAC during 3C fast charging of the battery module. Furthermore, the ICS demonstrated enhanced energy efficiency, performing 43.76% better than the FAC module.^[^ ^96^ ^]^

**Figure 10:**
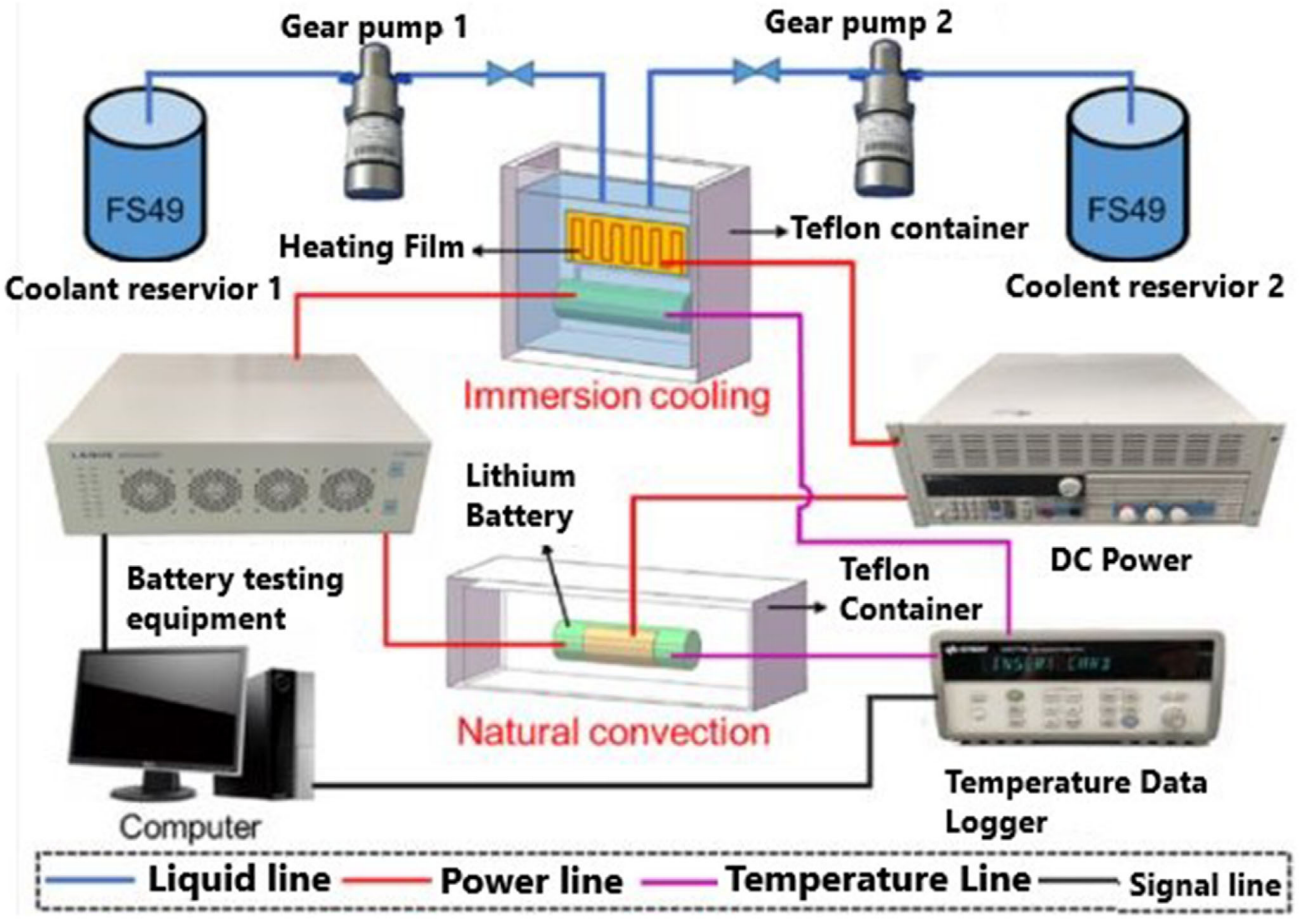
Representation of the reciprocating liquid immersion cooling system.^[^ ^97^ ^]^

**Figure** **10** shows Li et al. also looked into a reciprocating ICS for cylindrical batteries during fast charging utilizing FS49 fluorinated fluid. According to the study, the suggested approach considerably lowered the battery cell's *T*_max_ and *∆T* equated to natural convection cooling. The cooling approach ominously improved the battery's thermal performance and longevity by allowing the temperature to drop by up to 19.01 °C during the rest phase after fast charging.^[^ ^97^, ^98^ ^]^

Yao et al. demonstrated that the ICS provided excellent temperature control during the rapid charging of battery packs. This technique surpassed configurations with 10 and 20 mm gaps regarding the heat transfer coefficient (*h*_m_), achieving an impressive 1572.3 W m^−^^2^ K^−1^ with a 5 mm gap between battery cells and a *m*_f_ of 20 mm s^−1^. Additionally, incorporating a 48 mm‐tall disturbance structure reduced the surface Δ*T* of the cell by 44.3%, enhanced heat dissipation efficiency, and improved flow uniformity.^[^ ^99^ ^]^

**Figure 11:**
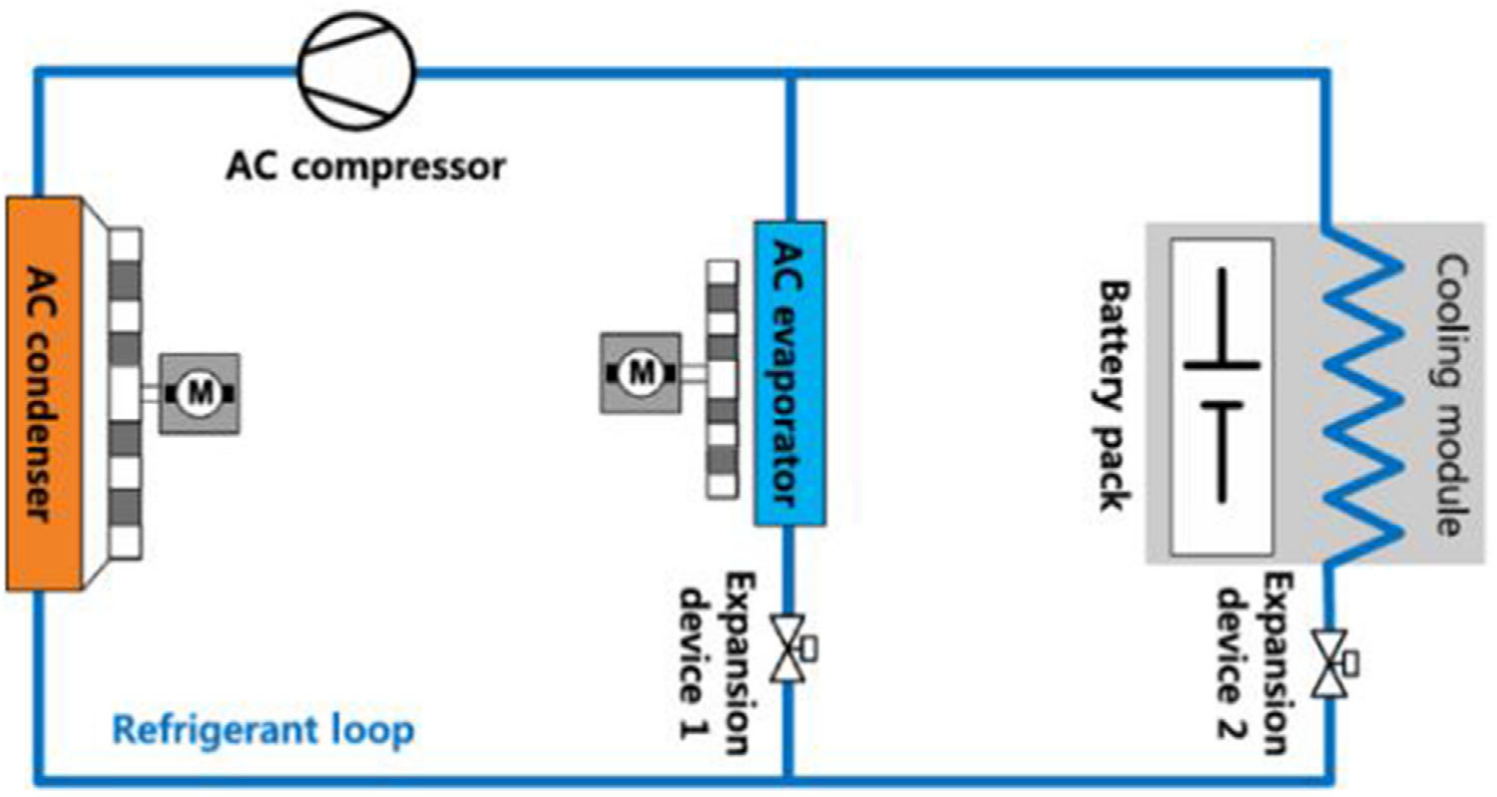
Battery cooling system schematic working on direct two‐phase refrigerant.^[^ ^100^ ^]^

As illustrated in **Figure** **11**, Hong et al. proposed a two‐phase refrigerant cooling method using R134a to enhance heat dissipation during fast charging. Their study demonstrated that this cooling system maintained the *T*_max_ below 45 °C during 2C fast charging. Additionally, two‐phase refrigerant cooling improved battery capacity by 16.1% and reduced internal resistance by 15.0% compared to traditional cooling systems over time.^[^ ^100^ ^]^

Williams et al.^[^ ^101^ ^]^ showed an experimental study on liquid ICS employing Novec 7000 dielectric fluid to assess its cooling efficiency during fast charging of a battery module. The findings indicated that ICS provided effective thermal management, particularly with two‐phase cooling. During 4C fast charging, the *T*_max_ and Δ*T* remained at 35 and 1 °C, respectively. Moreover, their investigation into cell spacing revealed that decreasing the distance between cells enhanced heat dissipation efficiency, leading to more controlled temperature rise and improved temperature uniformity across the battery module.

#### 3.2.2 Indirect Liquid Cooling

In contrast to liquid ICSs, liquid‐based indirect cooling systems are simpler to use. As illustrated in **Table** **2**,^[^ ^88^, ^102^, ^103^, ^104^, ^105^ ^]^ using ethylene glycol and water mixtures, typical coolants in these systems, leads to higher flow rates for the same pumping power due to their lower viscosity. As a result, the indirect‐contact method is often utilized, where the liquid flows through separate tubes, jackets, or cooled plates.^[^ ^106^, ^107^ ^]^ Due to its comparatively low price, liquid‐based indirect cooling is primarily used in BTMS of EVs.

**Table 2:** Physical and thermal features of different fluids for ICS.^[^ ^88^, ^102^, ^103^, ^104^, ^105^ ^]^

Material	Specific Heat Capacity [J kg^−1^ K^−1^]	Dielectric Constant	Density at 20 °C [g mL^−1^]	*k*_p_	Flash Point [°C]	Boiling [°C]
Mineral oil	1900		0.92	0.13	115	
Water–Glycol (1:1) mixture	3473	64.92	1.08	0.40	111	107
Silicone oil	1370	2.75	0.97	0.15	316	140
Poly‐alpha–olefins (Chevron Phillips)	2241		0.80	0.14	159	
Hydrofluoroethers (3 m Novec 7000)	1300	7.4	1.4	0.08	none	34

#### 3.2.3 Cold Plate

**Figure 12:**
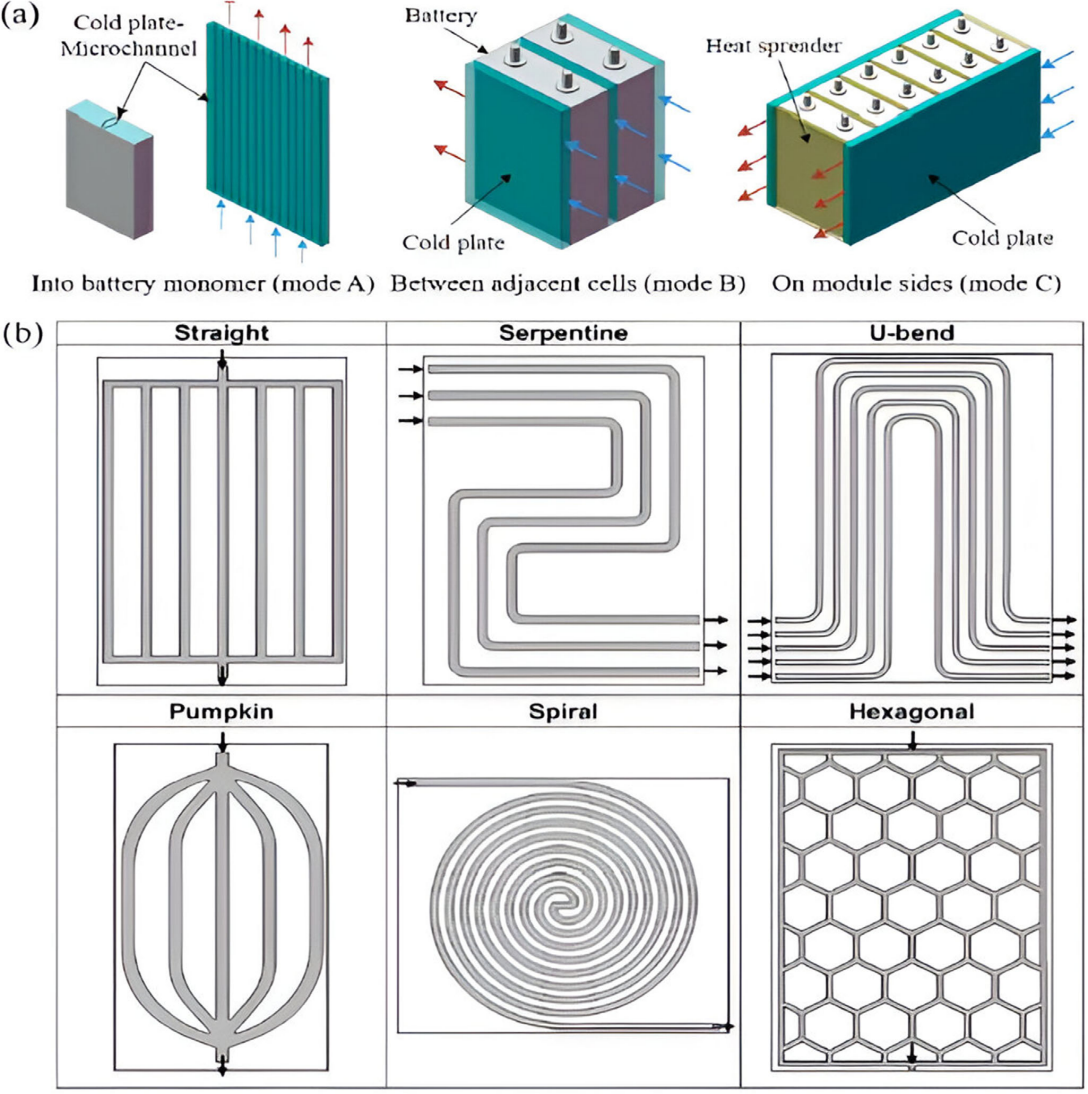
a) Cold plate arrangement;^[^ ^55^, ^105^ ^]^ b) Liquid channel arrangement for cold plate.^[^ ^113^, ^114^, ^115^ ^]^

A liquid cooling medium circulates through internal channels on a flat metal surface known as a cold plate.^[^ ^55^, ^108^, ^109^, ^110^, ^111^, ^112^ ^]^ The cold plate can be positioned in three different ways, as shown in **Figure** **12**a: committed to the edges of the battery module (mode C), positioned between consecutive batteries (mode B), or integrated within the battery monomer (mode A). Mode A necessitates that the channel dimensions be sufficiently small to fit inside the battery modules (Figure 12a) and that the jacket be chemically stable to prevent electrochemical corrosion.^[^ ^55^ ^]^ The cold plates are placed in between nearby batteries in mode B. The cold plate should have a narrow profile in order to maximize the battery system's energy density (Figure 12a).^[^ ^105^ ^]^ In mode C, the battery module's sides or bottom are usually in direct thermal contact with cold plates (Figure 12a).^[^ ^55^ ^]^ Heat spreaders enhance heat transfer between batteries and cold plates. Cold plates are frequently utilized in battery modules with prismatic cells rather than cylindrical ones because of their flat form. Cold plates ensure EV safety and compactness by providing essential sustenance for the cells and incorporating them with battery packs. All things considered, cold plates give the cells structural support and incorporate them into the battery pack, guaranteeing EV protection and compactness.

The performance of cold plate‐based BTMS can be enhanced by altering the channel design and liquid flow. The channel configurations frequently include straight, serpentine, U‐bend, pumpkin, spiral, and hexagonal shapes, as shown in Figure 12b.^[^ ^113^, ^114^, ^115^ ^]^

A cold plate with straight mini‐channels was generated by Huo et al.^[^ ^116^ ^]^ They investigated a 3D thermal model to examine the effects of flow direction, channel count, and inlet *m*_f_ on temperature increase and dispersion across the batteries during high‐rate discharge. They found that increasing the number of channels and the inflow *m*_f_ helped reduce the battery module's maximum temperature.^[^ ^116^ ^]^ Subsequent research revealed that five channels could increase the *m*_f_ while maintaining the battery temperature in the anticipated range.^[^ ^65^ ^]^ Furthermore, raising the channel's width from 3 to 6 mm led to a notable decrease in energy usage, encouraging energy efficiency and lowering emissions.^[^ ^117^ ^]^

Monika et al. further examined and contrasted the thermal performance of the six mini‐channel configurations previously discussed using a 3D numerical method. According to their findings, the pumpkin design provided a smaller pressure drop and required less pumping power, whereas serpentine and hexagonal channel shapes greatly enhanced temperature uniformity throughout the battery.^[^ ^113^ ^]^

**Figure 13:**
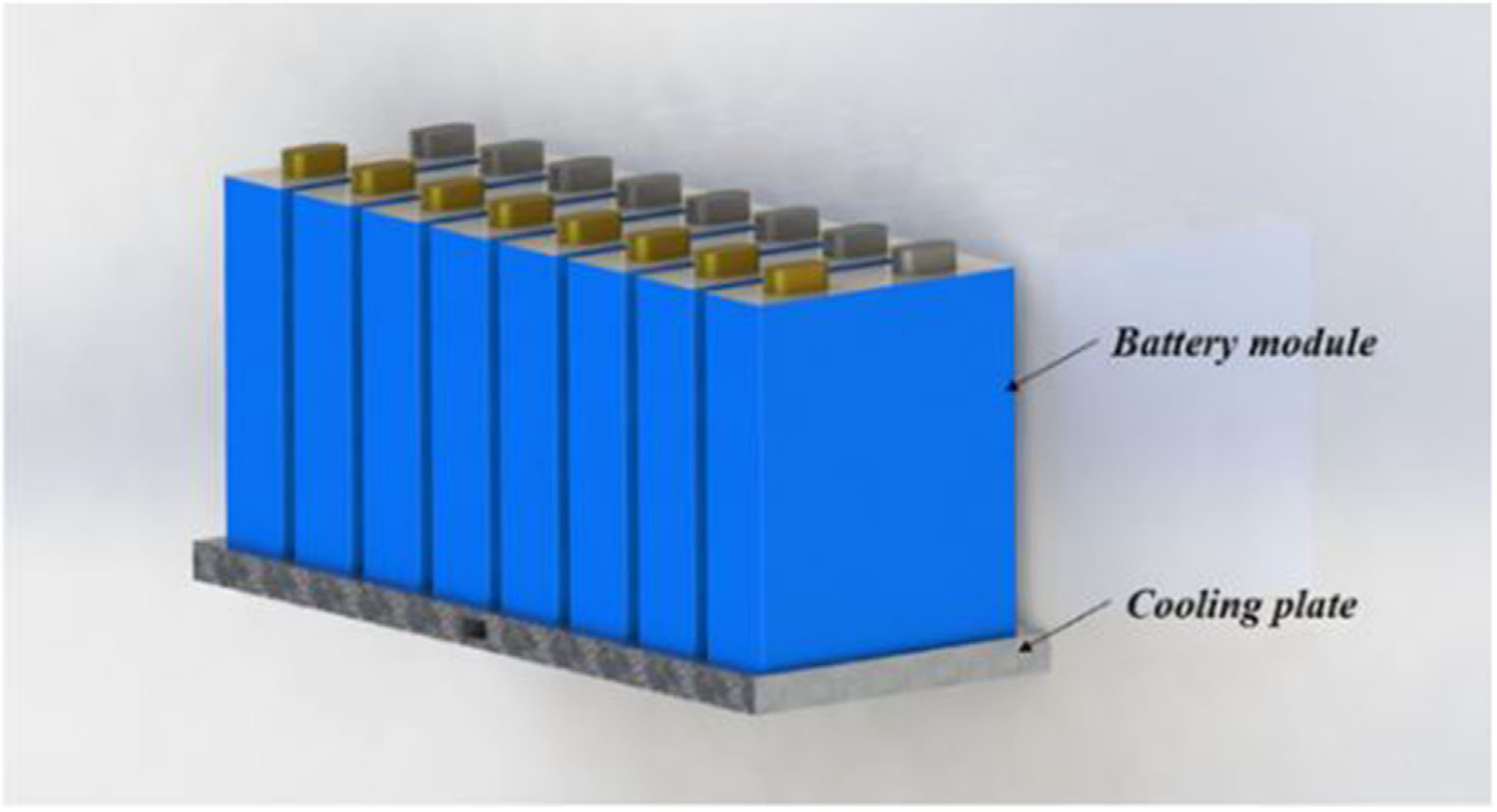
Parallel liquid‐cooled battery module.^[^ ^82^ ^]^

Chen et al. advocated a seven‐channel cooling plate with a parallel liquid cooling arrangement to reduce the prismatic battery module's charging time and improve temperature safety during rapid charging (**Figure** **13**). The previous research reveals the mini‐channel depth in a parallel liquid cooling system has the biggest effects on temperature uniformity, power consumption, and cooling efficiency—70.8%, 75.7%, and 86.1% of the total, respectively. The parallel liquid cooling system improved the volumetric energy density by 9.0%, the *T*_max_ by 2.1%, the Δ*T* by 23.7%, and the power consumption by 26.9% by optimizing the depth and width of the channels. The experimentally validated optimization model also demonstrated that with 2.5C fast charging, the battery module could maintain the *T*_max_, *∆T*, and energy consumption at 33.1 °C, 0.9 °C, and 17.29 J, respectively.^[^ ^82^ ^]^

Chen et al. suggested a quick LiB module charging cooling method using IDC with a mini‐channel topology. They suggested a neural network‐based regression model to expedite the design process for rapid charging and cooling systems, which lowers costs and time. Their experimentally validated optimal scheduling results revealed that after just 15 min of fast charging, the SOC of the battery module increased by 50%. This notable improvement in a brief time is essential for electric vehicle battery quick charging applications. Furthermore, the battery module's *T*_max_ and *∆T* were kept at 33.4 and 0.8 °C, respectively, even with a rapid charging rate of up to 2.5C, while the energy consumption for fast charging remained at 0.02 J.^[^ ^118^ ^]^

**Figure 14:**
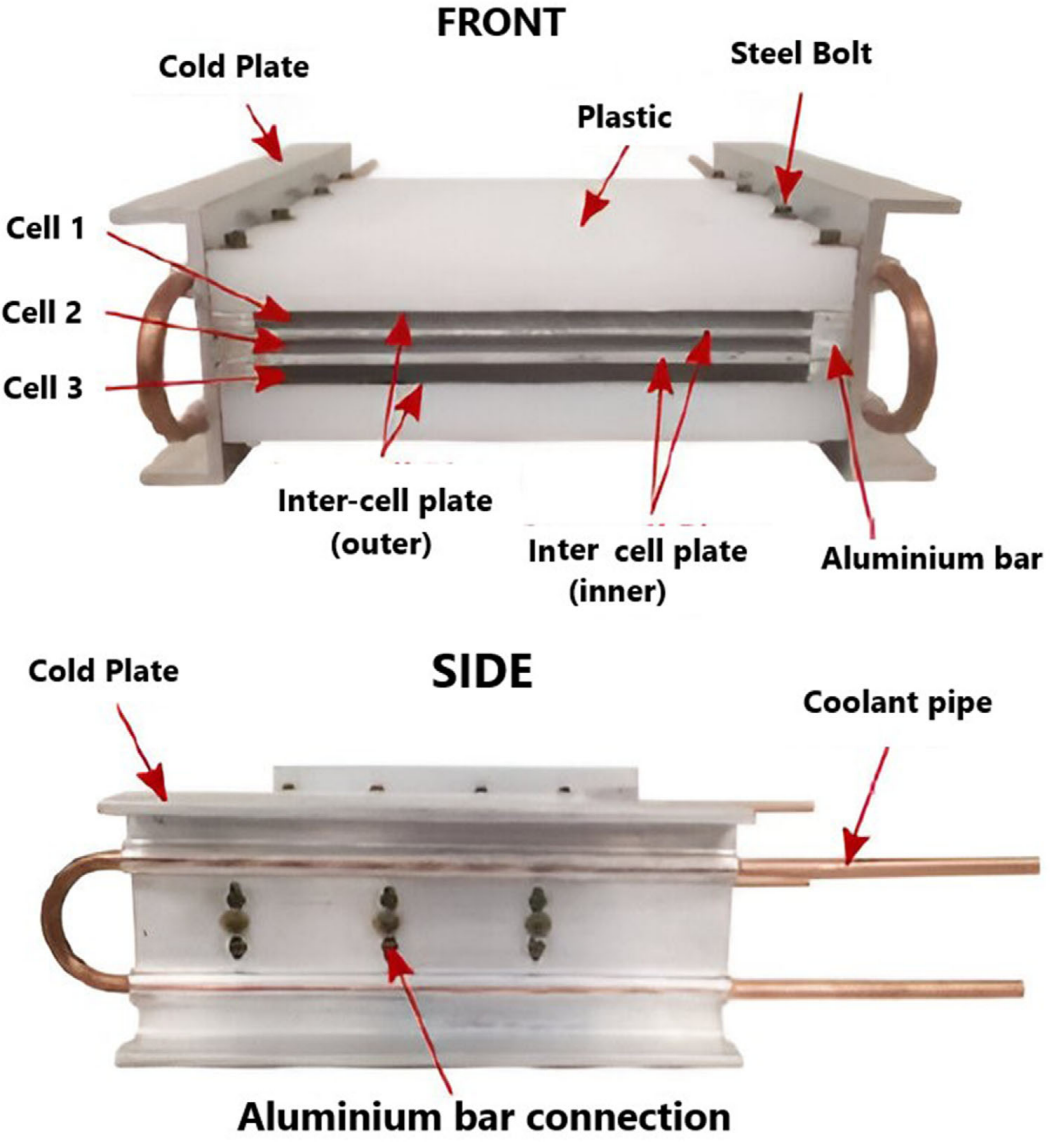
Cooling plate‐equipped 3P1S battery module model.^[^ ^119^ ^]^

To speed up the charging of battery modules using three Kokam NMC cells, Lempert et al. examined a 3‐compartment IDC technique (**Figure** **14**). According to the experimental results, the battery module's *T*_max_ was upheld at 22.3 and 28.5 °C for normal discharge rates of 1C and 3C, respectively. During fast charging at a 5C rate, the *T*_max_ was notably restricted at 34.6 °C.^[^ ^119^ ^]^

Wen et al. designed a double‐layer cooling device to regulate battery temperature during 3C rapid charging. Their investigation showed that the battery's *T*_max_ never rose above 45 °C even under high‐power and high‐temperature charging. It took 20 min for the SOC to charge from 30–80% and 28 min for the SOC to charge from 0–80%.^[^ ^120^ ^]^

Qin et al. proposed an exterior liquid cooling system with a 3‐sided cold plate design that controls temperature and prevents thermal runaway in order to protect the battery module during ultra‐fast charging. The cooling solution successfully reduced the battery module's *T*_max_ from 58–49 °C during 4C fast charging. Additionally, this technique ominously increased the driving range by 117 km when charging at a highly rapid 5C pace.^[^ ^121^ ^]^

To control the temperature increase and provide uniform heat dispersion in a cell‐to‐pack battery module during fast charging, Sun et al. employed a bottom liquid cooling plate structure in their models and experiments. Their findings demonstrated that the temperature of the battery was suggestively influenced by the cooling fluid's temperature and flow rate. The *T*_max_ decreased by 10.93% and 15.12%, respectively, under the same circumstances. Furthermore, their cooling method effectively kept the battery module temperature between 30 and 35 °C under New European Driving Cycle cycling situations, ensuring consistent temperature uniformity.^[^ ^122^ ^]^

Zhao et al. experimentally investigated two cooling strategies for battery modules under ultra‐fast charging settings. These include the inter‐cell cooling strategy, where coolant moves between cells, and the edge cooling technique, where coolant runs along the edges of the battery module. Their conclusions showed that the inter‐cell cooling method only resulted in a 4.1 °C temperature rise, whereas the edge cooling method produced a 14.2 °C temperature rise at a 5C ultra‐fast charging rate. This implies that the cooling efficiency of the inter‐cell cooling approach was higher. Furthermore, after several driving cycles and rapid charging at a 4C rate, the temperature rose by 12.2 °C using the edge cooling method and just 3.4 °C using the inter‐cell cooling method.^[^ ^123^ ^]^

**Figure 15:**
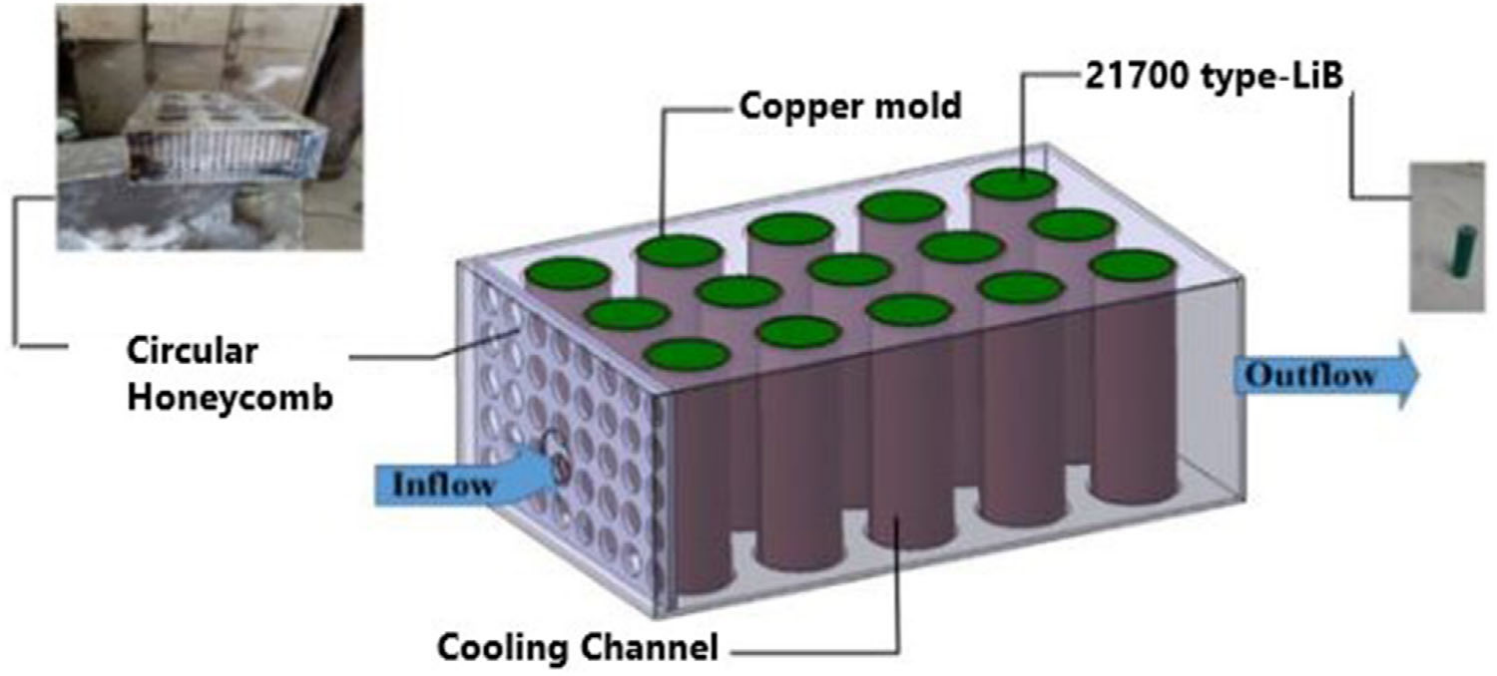
Indirect cooling‐based BTMS with AgO as nanofluid.^[^ ^124^ ^]^

Sarchami et al. assessed an indirect cooling approach for battery modules under fast charging and discharging situations using AgO‐NP and Cu mold construction, as shown in **Figure** **15**. The results showed that adding silver oxide nanoparticles to deionized water significantly reduced the battery module's *T*_max_ compared to a conventional water cooling system. Specifically, the *T*_max_ dropped by 7.3%, 11.1%, and 12% during fast charging/discharging with 1%, 2%, and 4% volume fractions of AgO, respectively. Under ideal circumstances, the *T*_max_ and *∆T* were maintained at 31.55 and 2.95 °C at a 5C charge/discharge cycle using 4% AgO‐NP with an intake velocity of 0.28 m s^−1^.^[^ ^124^ ^]^

## 4 Hybrid Cooling

### 4.1 Refrigerant‐Cooling

**Figure 16:**
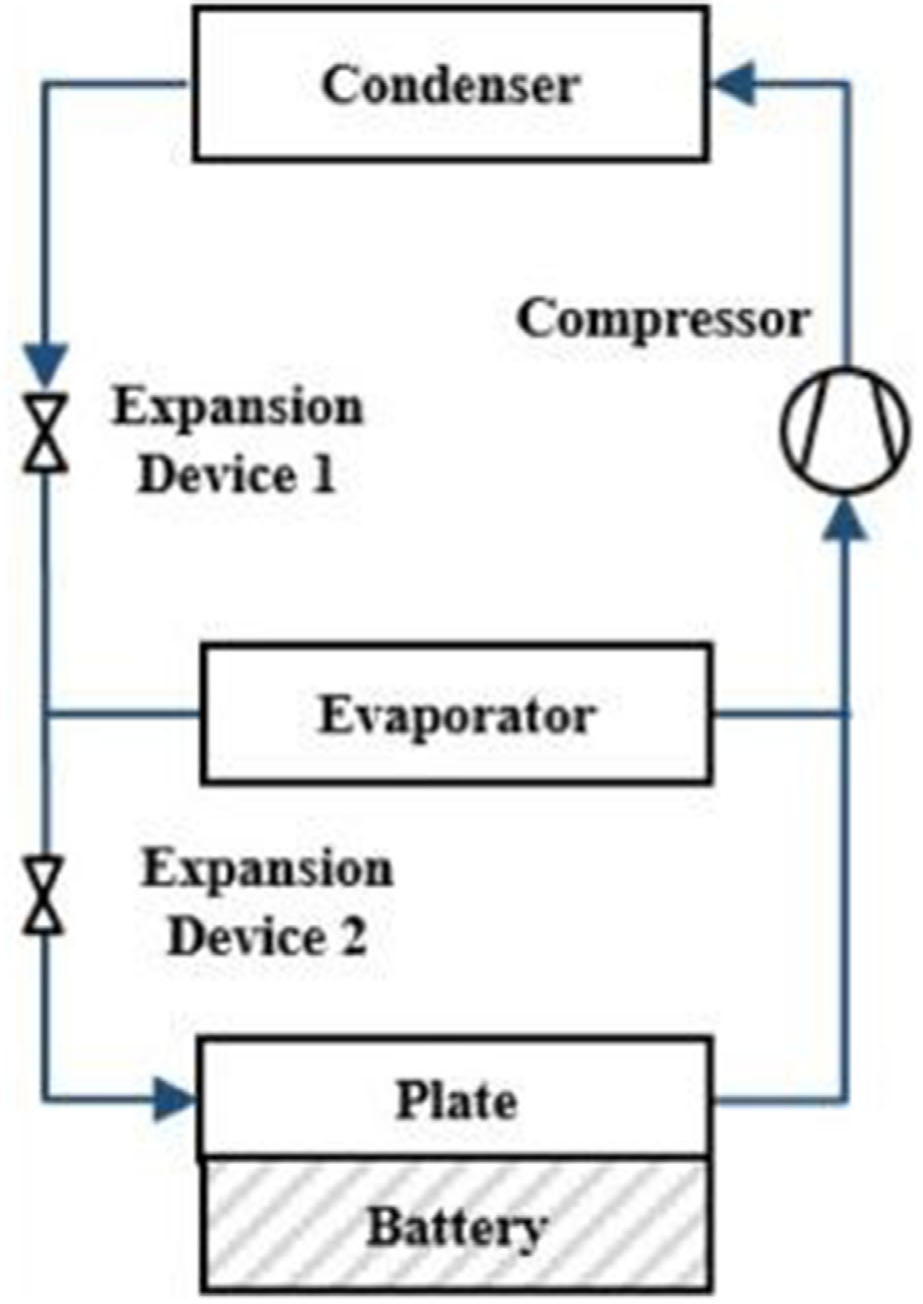
Schematic of the refrigerant cooling system.^[^ ^125^ ^]^

Refrigerant cooling is a subset of liquid‐based cooling. Still, it requires more intricate apparatuses and arrangements for operation,^[^ ^125^ ^]^ as illustrated in **Figure** **16**. For this reason, we have dedicated a separate section to discuss refrigerant coolants. The high cooling performance and ease of integration into electrified cars make this approach, a relatively new BTMS, a viable solution for electric and hybrid electric vehicles (EVs/HEVs).^[^ ^125^ ^]^ The concept is to adapt the VCC that EVs and HEVs utilize for air conditioning (AC) and cabin temperature adjustment to add a battery refrigerant cooling circuit. Studies have investigated the possibility of refrigerant cooling, even though the additional parallel evaporator in this method would result in higher power and energy usage.^[^ ^126^, ^127^, ^128^, ^129^ ^]^

One of the earliest studies in this area dates back to 2012 when Kruger et al.^[^ ^130^ ^]^ inspected a direct refrigerant cooling arrangement for battery temperature management. The research incorporated dynamic profiles to simulate hot and mild weather conditions while analyzing the refrigerant cycle's energy consumption. Findings revealed that under mild conditions, refrigerants R123a and R1234yf retained battery cell temperatures below 40 °C.^[^ ^131^ ^]^ In hotter climates, the *T*_max_ of the battery reached 42 °C with R123a and 48 °C with R1234yf, demonstrating the cooling efficiency of these refrigerants. However, the energy consumption of the refrigeration cycle is augmented by up to 11% in high‐temperature environments.

Modifying current AC and VCC arrangements has been the main focus of recent investigations on refrigerant cooling. Thomas Gillet and associates, for instance, rearranged the refrigerant distribution of a multi‐evaporator AC system for EVs.^[^ ^132^ ^]^ At high load conditions, the cooling needs forced the evaporators to run at different capacities, sending super‐heated refrigerant from the first evaporator to the second. Normally, this system operates with two evaporators working together dynamically. Gillet et al.^[^ ^132^ ^]^ aimed to ascertain by experimentation the ideal thermal interaction between the refrigerant distribution and the evaporators. After comparing various experimental configurations, they found that the peak in the outlet air temperature at the start of the battery cooling process could be reduced by gradually opening the electronic valve and increasing the compressor speed.

Additional research has investigated alternative coolants to replace conventional R134a. For example, van Gils et al.^[^ ^133^ ^]^ and An et al.^[^ ^134^ ^]^ introduced a novel BTMS utilizing boiling heat transfer with HFE 7000 as the cooling medium. Their findings showed that this cooling fluid offers excellent insulation properties and superior thermal performance compared to air, contributing to better thermal uniformity in the cell during boiling.

**Figure 17:**
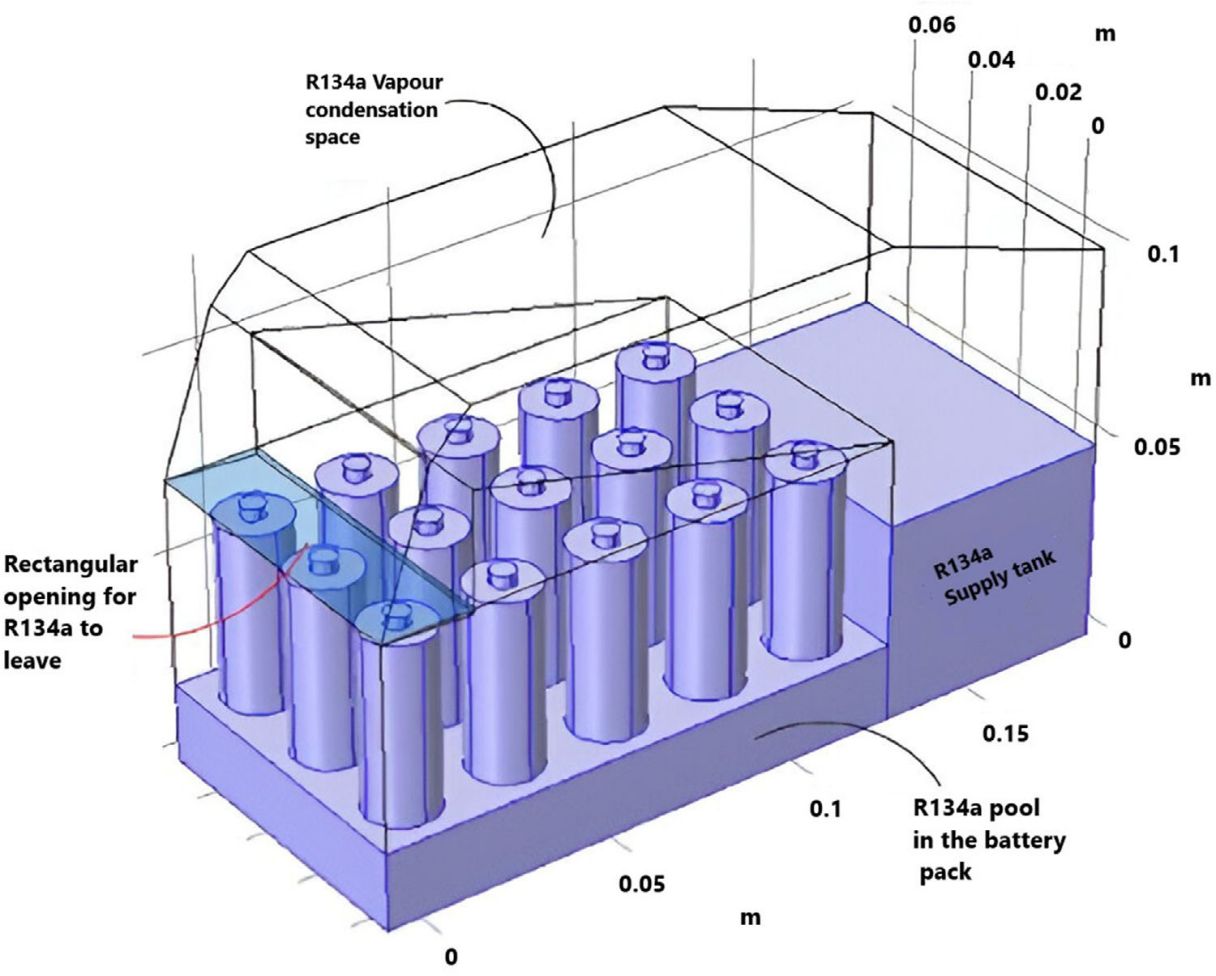
Schematic of refrigerant‐cooling system for a battery module.^[^ ^135^ ^]^

Furthermore, several studies have concentrated on simulating 3D models for refrigerant‐based cooling systems due to the relatively low computational complexity. In,^[^ ^135^ ^]^ Al‐Zareer et al. planned a refrigerant cooling arrangement by means of CFD. As illustrated in **Figure** **17**, the system utilized the phase‐change fluid R134a and partly immersed a 15‐cell battery module in a designated pool. The generated R134a vapor, which absorbed heat from the battery, was recirculated through a return channel after collecting from the top. Despite its straightforward design, the system exhibited competitive performance associated with conventional cooling methods, such as air or liquid‐based systems. The refrigerant cooling system demonstrated efficiency under high‐cycle loads (5C or 60A), successfully keeping the battery temperature below 30 °C while requiring contact with only 40% of the battery surface. Furthermore, using CFD to optimize the refrigerant cooling design led to a useful model, allowing for the proper selection of two‐phase fluids, as seen in other studies on refrigerant selection.

In conclusion, refrigerant‐based cooling systems show promise as a viable BTMS, though they require additional thermal components that can significantly increase power consumption. Moreover, extra heaters or a separate heat pump system would need to be joined for winter applications, which can pose challenges in electrified vehicle applications. However, it is expected that further research will continue to explore refrigerant‐based BTMS, with an emphasis on simplifying the system and reducing integration complexity and costs.

### 4.2 PCM‐Cooling

Due to the bulky nature and added expense of conventional cooling methods—often involving components like fans, piping, and other hardware—there has been growing interest in exploring passive thermal storage systems as an alternative approach to BTMSs.

**Figure 18:**
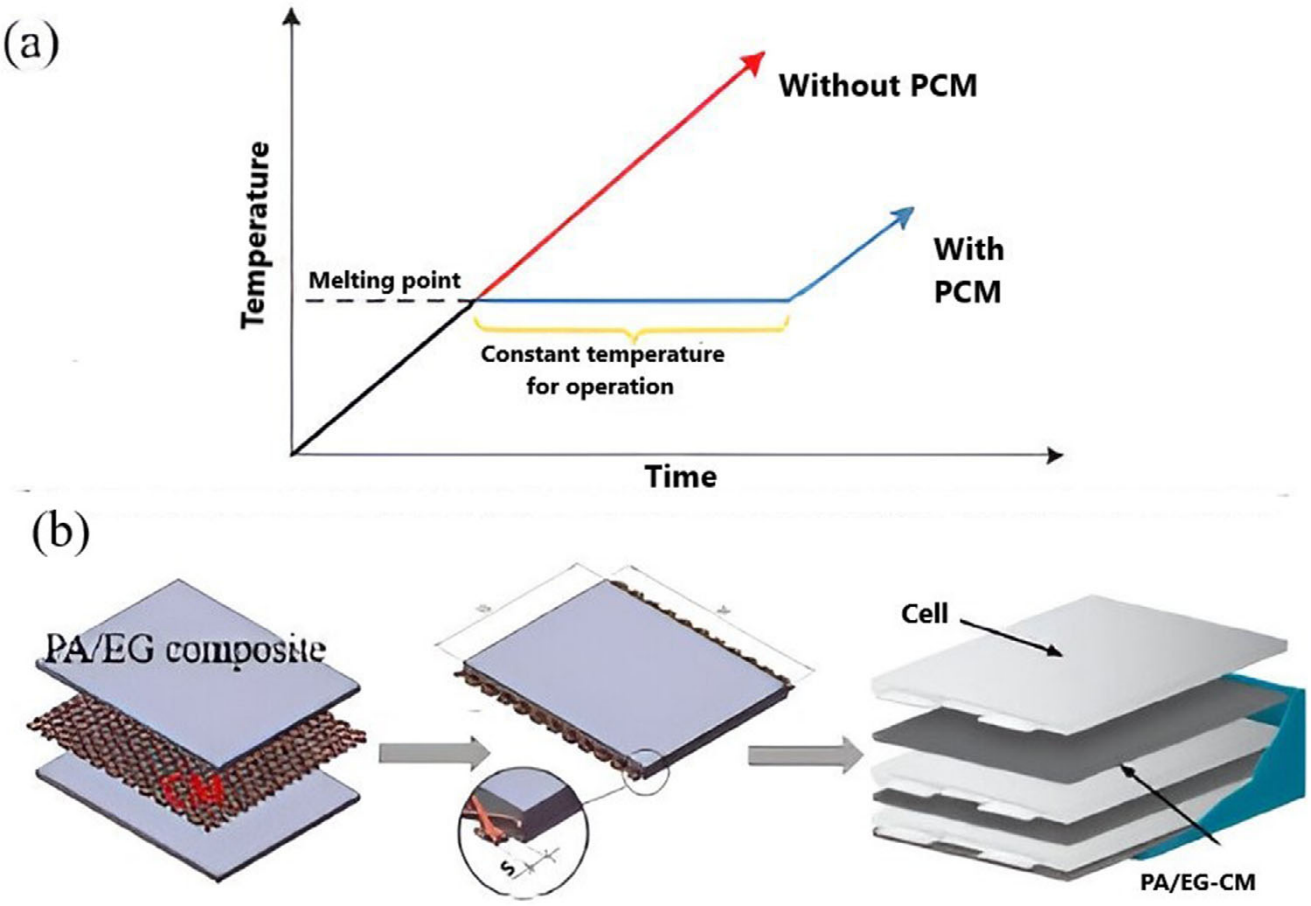
a) Temperature properties of the PCM‐based system;^[^ ^55^ ^]^ b) the arrangement of the PA/EG‐CM composite and its use in BTMS.^[^ ^138^ ^]^

PCMs are materials that store or release heat during phase transitions. As a ground‐breaking solution for thermal management, PCMs can captivate substantial latent heat during melting, helping to uphold a stable temperature near the phase transition point for a prolonged period (**Figure** **18**a).^[^ ^55^, ^136^ ^]^ PCMs with melting points ranging from 20 to 60 °C are commonly selected to align with the operating temperatures of LiBs.^[^ ^136^, ^137^ ^]^ Despite their ability to enhance temperature homogeneity in large‐scale batteries under high‐rate discharge conditions, PCMs still face challenges such as low *kp*, significant volume expansion, flammability, poor structural stability, and the risk of leakage when melted.^[^ ^25^ ^]^

Shape‐stabilized PCMs, which contain PCM (dispersed phase) and other materials as stabilizing agents, have been proposed as a solution to these restrictions.^[^ ^138^ ^]^ It has been discovered that adding frameworks or additives with different characteristics can improve form retention, absorb liquid PCMs, and increase *kp*.^[^ ^140^ ^]^ Lv et al. established a composite PCM enhanced with nano‐silica (NS), offering improved resistance to leakage and volume changes for BTMS. By incorporating a small amount of NS into PA, they showed that the nanoscale pores of NS, fluctuating from 30 to 100 nm, effectively absorb liquid PA. This prevents leakage, enhances uniformity, and minimizes volume fluctuations during phase transitions.^[^ ^141^ ^]^

Metal particles,^[^ ^142^ ^]^ metal foams,^[^ ^143^ ^]^ carbon fibers,^[^ ^134^ ^]^ graphene,^[^ ^136^ ^]^ and carbon nanotubes^[^ ^140^ ^]^ are some of the materials that have been investigated to recover the *kp* of PCM‐based BTMS further^[^ ^140^ ^]^ Shirazi et al. created various PA nanocomposites by combining graphene, fullerene, and carbon nanotubes. Goli et al. created a composite using PA mixed with 1 wt% graphene, which exhibited a 60‐fold increase in conductivity equated to traditional PA.^[^ ^144^ ^]^ Wu et al. presented novel PA/EG composites strengthened by pyrolytic graphite sheets (PGS), in which EG forms a primary thermal conductive network (TCN) and absorbs the liquid PCM without leaking. By creating a supplementary TCN, PGS, which is affixed to the battery module's sidewalls, enhances thermal uniformity.^[^ ^145^ ^]^ Additionally, they suggested a PA/EG composite (PA/EG‐CM) improved by copper mesh (CM) for BTMS (Figure 18b).^[^ ^139^ ^]^ While CM acts as a framework to increase the module's strength and *kp*, EG's porous nature enables it to absorb liquid PA and stop leaks during phase shifts. When equated to the PA/EG plate without CM, this composite showed better heat conduction and temperature homogeneity.^[^ ^139^ ^]^ **Table** **3** provides specifics on these improved PCMs' better features.

**Table 3:** The improved characteristics of PCMs used in BTMS.^[^ ^8^, ^130^, ^139^, ^140^, ^141^, ^142^ ^]^

PCM/*kp*	Ratio of Composite [% wt]	Latent Heat of PCM without/with Additives [kJ kg^−1^]	Composites *kp*	Additives/*kp*
Paraffin/0.2	7/5.5/30	−/77.8	3.5	Silicon/‐ and expanded graphite/4–100 and polyethylene/‐
Paraffin/0.31	12	133.1/90	0.46	Graphite powder/2–90
Paraffin/0.2697	6.25	—/—	4.676	Expanded graphite/4–100
Paraffin/0.25	5 (vol%)	—/—	0.6	Graphene/3000
Paraffin/0.25	5 (vol%)	—/—	2.5	Carbon nanotubes/3000
Paraffin/0.21	0.69	242/—	0.42	Carbon fiber/50
Hexadecane/0.15	—	236/167	1.25	Aluminum particles
Erythritol/0.733	34 (vol%)	—/—	4.72	Nickel particle/90.3

A widely adopted method in numerous studies involving electrochemical solutions with PCM is modifying the internal composition of the PCM by incorporating a module that enhances *k*_p_ or other beneficial properties. For example, Abid Hussain et al.^[^ ^146^ ^]^ conducted an experimental study where they developed graphene‐coated nickel (GcN) foam infused with PA to improve overall conductivity. This modification resulted in a conductivity 23 times higher than that of pure paraffin.

The development of GcN demonstrated additional advantages, including an increased melting point, a lowered freezing point, and a 30% reduction in latent heat. Compared to traditional PCMs, GcN effectively reduced the temperature rise of the battery system by 17%. Other research efforts have focused on experimentally formulating PCM with metallic elements such as Cu and Al. These materials are intended to augment *kp* and expand the specific surface area, facilitating more efficient heat transfer. For example, Mallow et al.^[^ ^147^ ^]^ examined the effect of Cu foam on PCM and reported promising outcomes.

**Figure 19:**
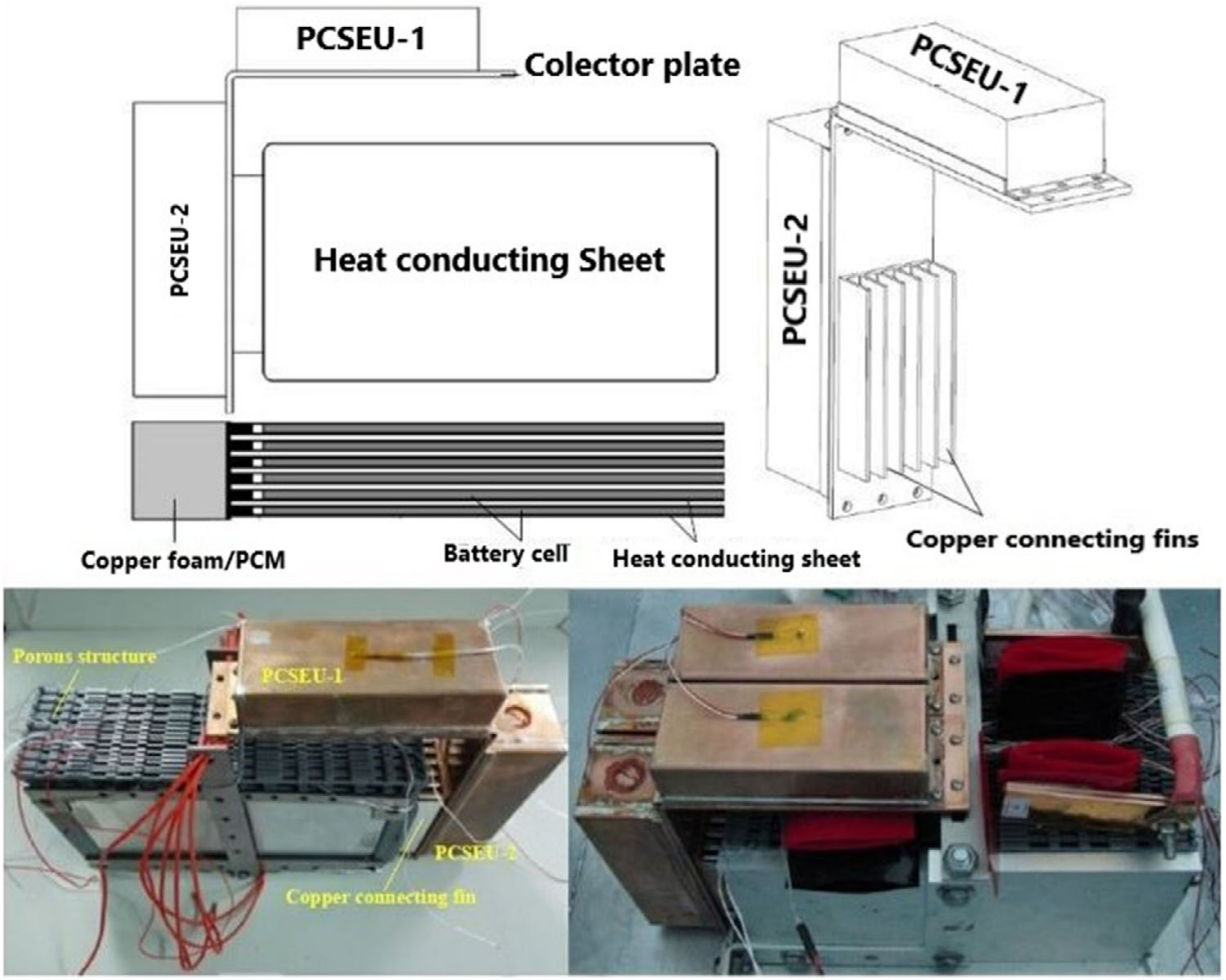
Graphical view of the TMS used in ref. [149].

Additionally, studies have explored the incorporation of metallic constituents for physical integration into battery systems.^[^ ^148^ ^]^ For example, Xiaoming Wang et al.^[^ ^149^ ^]^ developed a novel passive TMS utilizing PA for a LiB module. As illustrated in **Figure** **19**, this system includes a phase change energy storage unit (PCSEU) consisting of Cu foam and PA, which is linked to the pouch cell electrodes through Cu conducting fins. This innovative design allows for the integration of additional PCMs while ensuring that the PCSEU does not make direct contact with the cell. The proposed system successfully supported up to three cycles of 4C (40 A) charge and discharge at 35 °C, maintaining the *T*_max_ of the battery pack below 52 °C. Additionally, numerous studies have explored PCM modelling and numerical solutions to determine the optimal PCM design for specific battery systems.^[^ ^150^, ^151^, ^152^ ^]^

**Figure 20:**
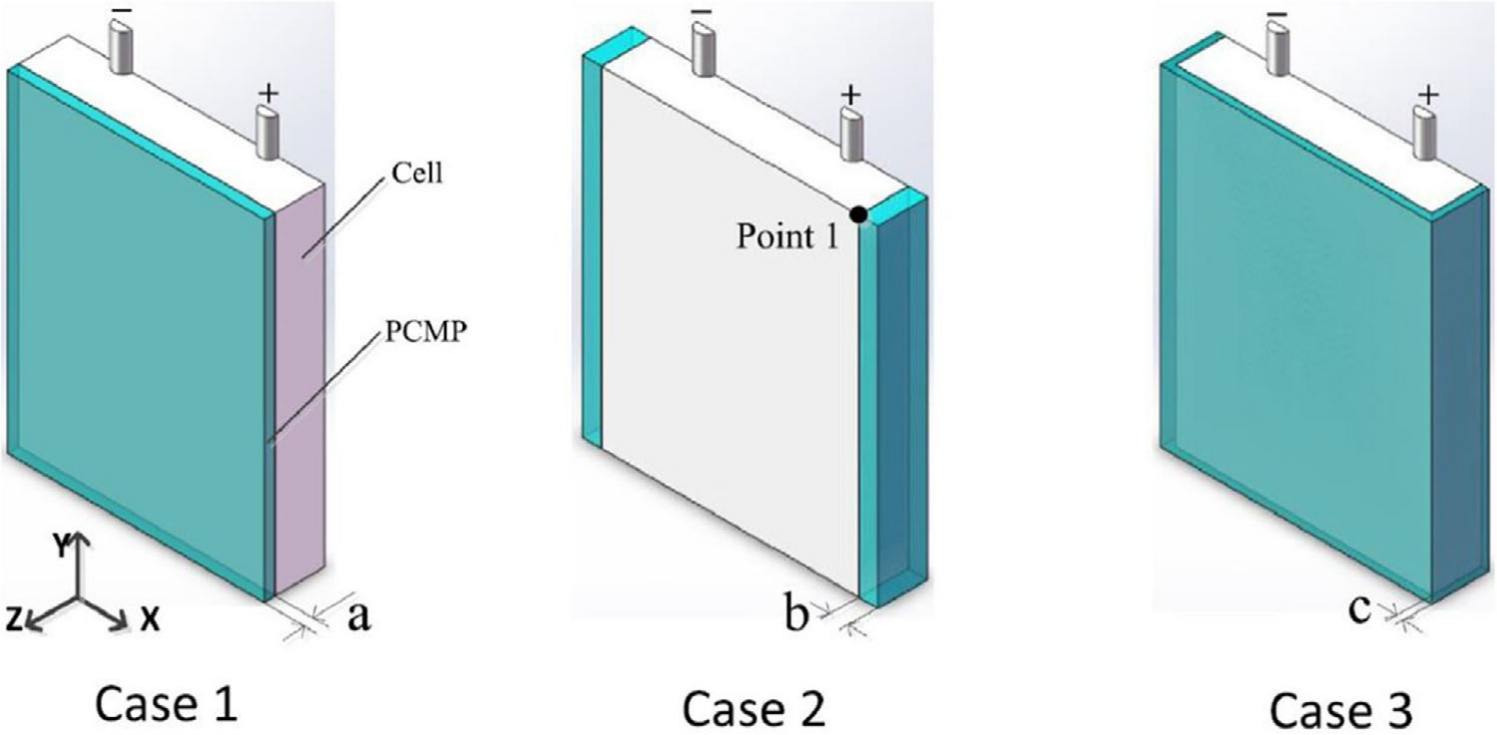
PCM plate (PCMP) configurations with three casings used in ref. [153].

In a similar study, Weixiong Wu et al.^[^ ^153^ ^]^ created a 3D thermal model for a prismatic battery and enhanced its performance by employing three distinct shape‐stabilized PCM plate designs, as illustrated in **Figure** **20**. The PCMs selected for the study were PA and expanded graphite, with the volume of the PCMPs being consistent across all three configurations.

The configuration in which the PCMP encased the cell (case 3) provided the most effective thermal performance, as it had a larger contact area with the cell. Additionally, using numerical simulations, the authors were able to assess the impact of PCM thickness on cooling efficiency. Their results showed that when the thickness was less than 2.08 mm, the temperature distribution experienced considerable variation.

To minimize the thermal gradient within the battery pack and address the challenge of balancing high heat storage capacity with low *kp* (such as paraffin wax with 0.25 W m^−1^ K^−1^), various methods have been explored to create composite PCMs. These approaches involve: 1) incorporating a metal matrix into the PCM, 2) impregnating porous materials,^[^ ^154^, ^155^, ^156^, ^157^, ^158^ ^]^ 3) adding high *kp* materials to paraffin,^[^ ^159^, ^160^ ^]^ and 4) designing latent heat TES systems with both finned and unfinned structures.^[^ ^161^, ^162^ ^]^ The *kp* of composite PCMs, such as PCM/graphite matrix, ranges from 3 to 16.6 W m^−1^ K^−1^.^[^ ^161^, ^162^, ^163^ ^]^ Examples of composite PCM applications in vehicles are summarized in **Table** **4**. However, a decrease in latent heat storage capacity is frequently the result of an increase in *kp*. Maintaining the proper *kp* ratio (*k*_pcm_:*k*_c_) between the PCM and battery cells is essential for achieving peak performance.^[^ ^164^, ^165^, ^166^ ^]^ Furthermore, PCM characteristics, including stability, non‐toxicity, non‐flammability, and non‐explosiveness, are essential for guaranteeing battery safety. Thus, to endure thermo‐mechanical impacts while in use, a strong and sturdy PCM‐based battery module is needed. Alrashdan et al.^[^ ^167^ ^]^ thoroughly investigated the thermo‐mechanical properties of the paraffin wax/expanded graphite composite PCM for LiB. Their findings indicated that increasing the % of paraffin wax improved tensile, compressive, and burst strengths at room temperature, although the effect was less pronounced at elevated temperatures.

**Table 4:** Composite PCMs applicable for vehicles.

Ref. [s]	Applications	PCM characteristics
Khateeb et al.^[^ ^164^ ^]^ (electric scooters)	For a large LiB pack targeting at HEV/EV uses.	PCM/aluminum foam: $\begin{array}{c} k_{eff}=k_{pcm}\varepsilon +\left(1-\varepsilon \right)k_{al}\\ \rho_{eff}=\rho_{pcm}\varepsilon +\left(1-\varepsilon \right)\rho_{al}\\ C_{p}=C_{p}\varepsilon +\left(1-\varepsilon \right)C_{p,\mathrm{al}},h=195\;kJ\;kg^{-1} \end{array}$
Sabbah^[^ ^165^ ^]^		PCM/graphite matrix: 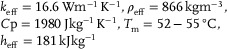
Kizilel et al.^[^ ^168^ ^]^		PCM/graphite matrix: 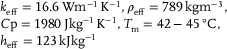
Li et al.^[^ ^143^ ^]^		PCM/copper metal foam: *k*_eff_ = 11.33/6.35/0.8 Wm^−1^ K^−1^ from different samples
Duan^[^ ^169^ ^]^	For a comparison study based on a simulated single cylindrical battery cell	PCM 1 provided by the Glacier Tek Inc.: 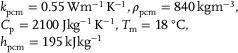
Rao^[^ ^166^, ^170^, ^171^ ^]^	For cylindrical NiMH and rectangular lithium‐ion batteries	PCM/graphite matrix: ρ_pcm_ = 910 kgm^−3^,*T*_m_ = 18 ^°^C

However, the promising performance of PCM‐based thermal systems has so far been demonstrated primarily under controlled laboratory conditions, far from real‐world deployment. Significant progress is still needed before these systems can be practically implemented. Additionally, with upcoming fast‐charging standards, PCM solutions' current heat absorption and dissipation capabilities are insufficient. As a result, integrating supplemental cooling techniques alongside PCMs should be considered essential to effectively manage temperature and avoid critical issues such as thermal runaway.

### 4.3 Heat Pipe

Heat pipes are widely recognized for their efficient cooling and thermal management in various industrial applications,^[^ ^172^ ^]^ though their use in BTMS has not yet been fully explored.^[^ ^173^, ^174^, ^175^ ^]^ Due to the inherent limitations of PCMs, such as low thermal conductivity, volume expansion, and slow response times, integrating heat pipe (HP) technology into TMS has emerged as an effective solution. Heat pipes offer a promising passive TMS alternative. They typically consist of a sealed tube divided into three sections: an evaporator, an adiabatic region, and a condenser. A working fluid within the pipe allows efficient thermal energy transport via phase change and capillary action.

Heat pipes can effectively transmit heat to cool or heat a battery as needed while using little power, much like the passive method provided by PCMs. Through capillary action in a porous wick lining, the HTF is passively returned to the evaporator, and the latent heat of vaporization transfers heat from the evaporator to the condenser. This cycle allows heat to be continuously absorbed and emitted.

**Figure 21:**
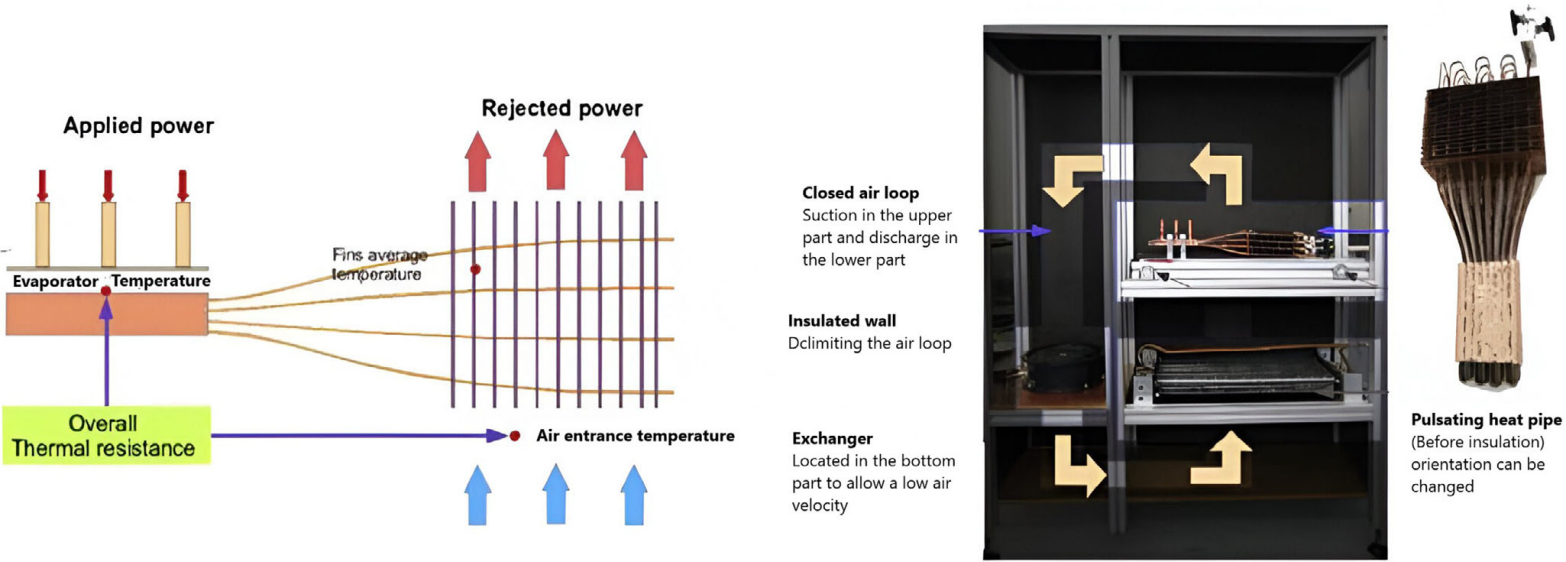
Pulsating heat pipe cooling a HEV lithium‐ion battery pack.^[^ ^181^ ^]^

**Figure 22:**
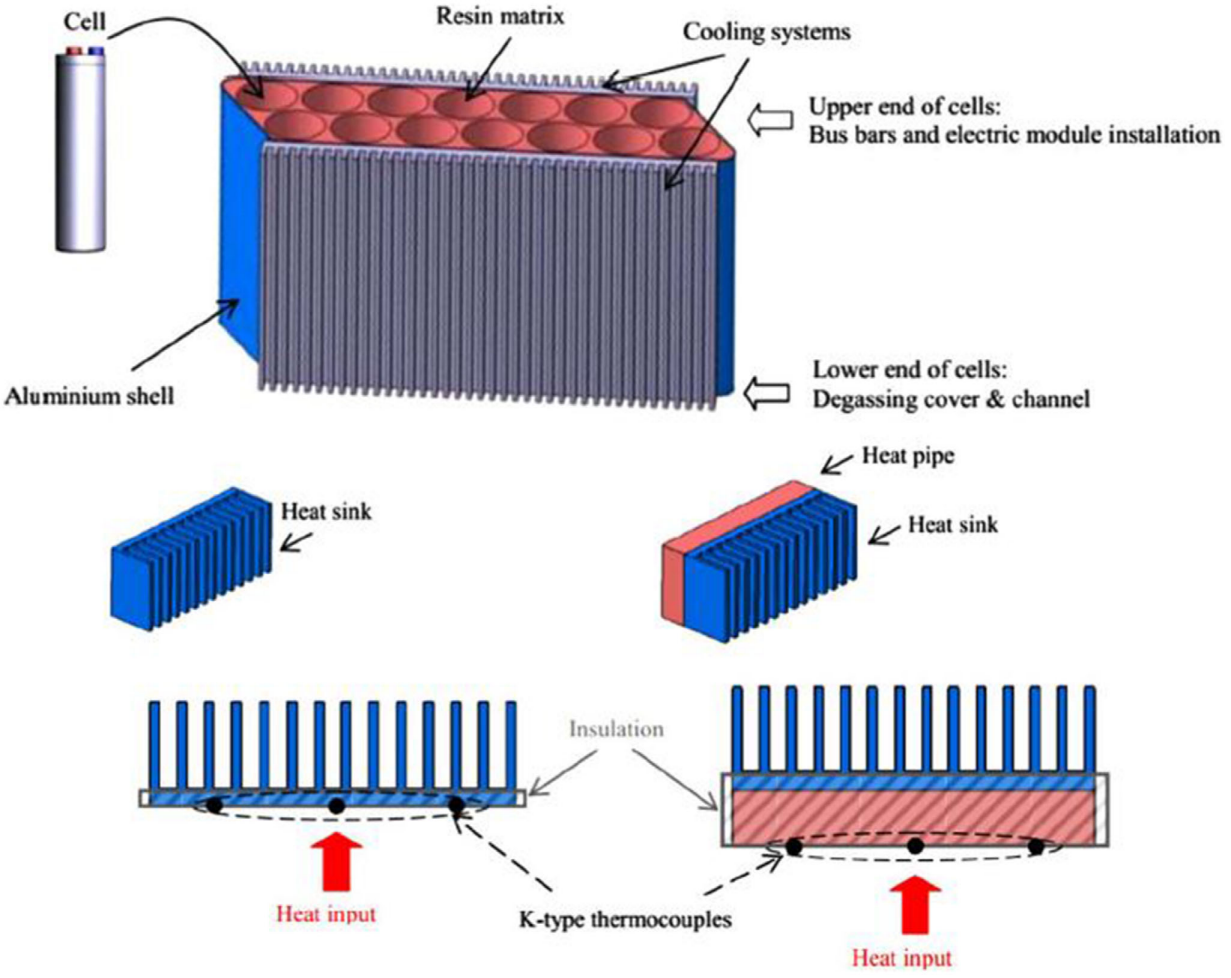
Flat heat pipe cooling a HEV LiB pack.^[^ ^182^ ^]^

Early research investigated the use of heat pipes in conjunction with air conditioning. For instance, Swanepoel^[^ ^176^ ^]^ suggested employing pulsing heat pipes (PHPs) to regulate HEV components and manage the thermal management of Optima Spirocell lead–acid batteries. According to simulation and experimental results, a well‐designed PHP system needed heat pipes with a diameter of <2.5 mm and ammonia as the HTF. Wu et al.^[^ ^177^ ^]^ recommended using heat pipes with Al fins to cool large‐scale lithium‐ion batteries; nevertheless, they encountered issues with heat dissipation at the battery's center without a cooling fan at the condenser. A loop thermosyphon cooling technique that integrated air cooling and heat pipes was used by Jang and Rhi.^[^ ^178^ ^]^ Barantsevich and Shabalkin^[^ ^179^ ^]^ assessed ammonia axial‐grooved heat pipes for thermally controlling a solar battery drive, whereas Park et al.^[^ ^180^ ^]^ optimized a loop heat pipe system to cool lithium‐ion batteries in a military aircraft. Burban et al.^[^ ^181^ ^]^ tested an unlooped PHP (with a 2.5 mm inner tube diameter) in combination with an air heat exchanger to cool electrical equipment in hybrid cars (**Figure** **21**). They used a hybrid driving cycle (New European Driving Cycle) to test various heat pipe HTF, air velocity, and inclinations in both steady‐state and transient conditions. Tran et al.^[^ ^182^ ^]^ proposed using a flat heat pipe to cool HEV lithium‐ion batteries under both forced and natural convection, highlighting the efficiency under different heat pipe orientations (**Figure** **22**).

**Figure 23:**
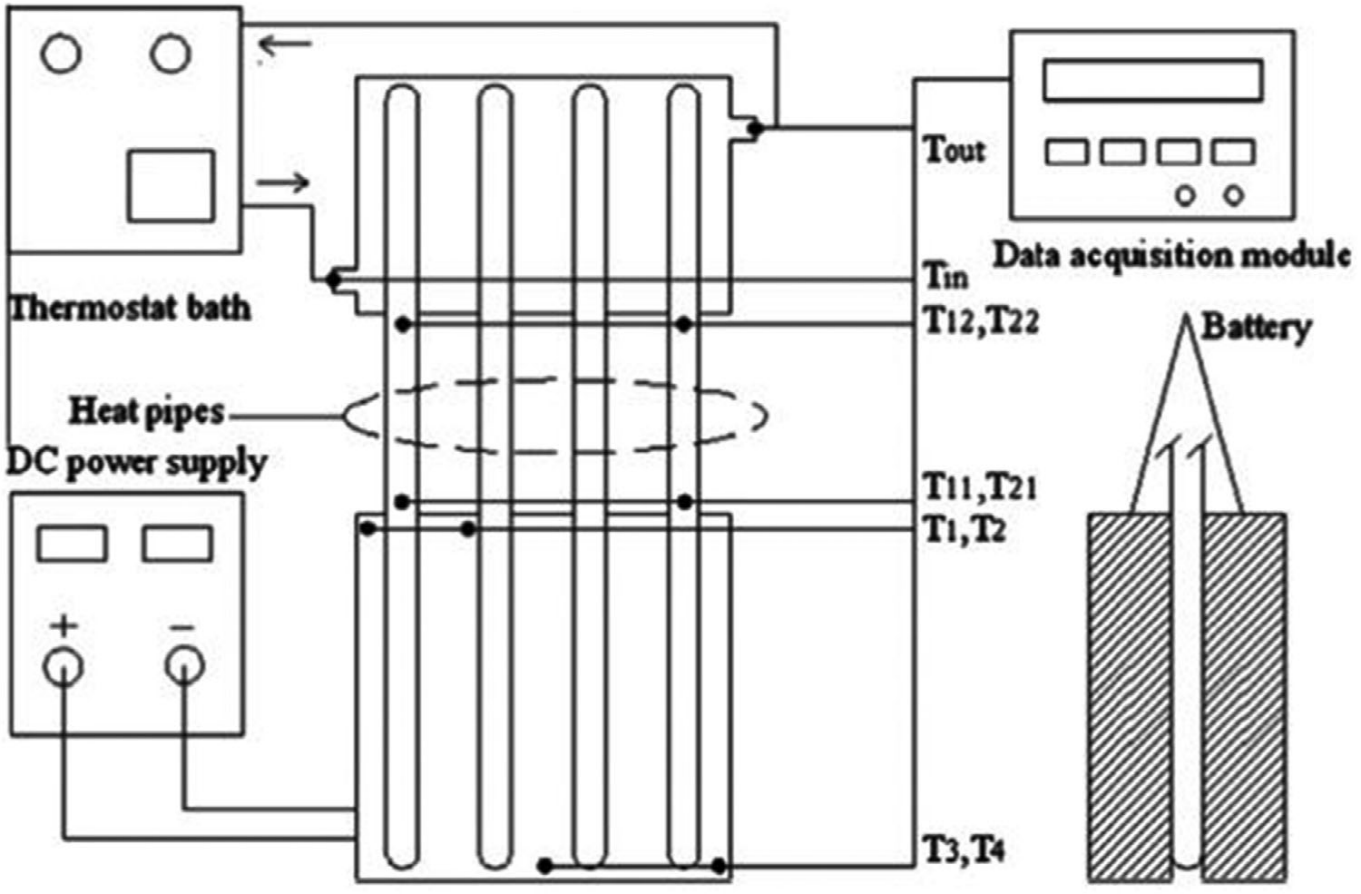
Cylindrical flattened heat pipe cooling for a118*63*13mm8Ah LiFePO_4_.^[^ ^183^ ^]^

In their experimental study of a heat pipe‐based cooling system for commercial prismatic LiFePO_4_ batteries, Rao et al.^[^ ^183^ ^]^ illustrated the coupling of heat pipes with liquid cooling. The condenser of the heat pipe was cooled in their experiment using a water bath maintained at 25 ± 0.05 °C (**Figure** **23**). The findings imply that heat pipes may be able to manage higher heat flow more successfully than conventional heat sinks; nevertheless, more research is necessary to determine whether incorporating heat pipes into car batteries is feasible. Cost, weight, mass production capability, material compatibility, transient behavior under high‐frequency and large‐amplitude variable power inputs, and efficiency degradation due to vehicle shocks and vibrations are all significant factors. Heat pipe‐based BTM is still in its infancy compared to air and liquid‐based BTM systems.

Therefore, it is recommended that research be extended to the pack level to fully understand the impact of thermal accumulation under various cycling conditions.

## 5 Other Recent Method

A battery management system performs several tasks to track a battery's condition and ensure it operates within a safe range.^[^ ^184^, ^185^ ^]^ One of its key roles is assessing the SOC, which reflects the battery's remaining energy. The SOC of a battery is estimated using indirect measurement of voltage, current, and temperature.^[^ ^186^, ^187^ ^]^ Common methods for estimating SOC include OCV readings, Coulomb counting, EKFs, and approaches that utilize artificial intelligence algorithms.^[^ ^187^, ^188^ ^]^

OCV method estimates the SOC using the direct correlation between the OCV and the SOC, under a 1:1 ratio, without real‐time estimation because it requires the battery to rest for an OCV measurement. Coulomb counting calculates the SOC by integrating the current during charging or discharging processes, which results in real‐time SOC estimation with minimal data loss. However, it relies heavily on accurate initial SOC values and accumulates errors over time due to inaccuracies in the measured current. Advancement of deep learning and data‐driven approaches for SOC estimation using measurable battery data have been explored.^[^ ^189^ ^]^ These methods do not require complex battery models or deep electrochemical understanding, as they learn the relationship between the battery data and the SOC. While a large dataset is needed for training, this technique eliminates the need to account for changes in battery parameters or the nonlinear effects of ageing and operating conditions.^[^ ^190^ ^]^

### 5.1 Recurrent Neural Network

The recurrent neural network (RNN) is one such deep learning technique that features a loop within its hidden layers, allowing it to retain sequence information from the current time step. This makes it effective at mapping battery parameters like voltage, current, and temperature to the SOC. However, when training on long time‐series data from battery charge/discharge cycles, the RNN model is prone to the vanishing gradient problem. This issue hampers its ability to capture long‐term dependencies in the data, leading to reduced accuracy in SOC estimation.^[^ ^191^ ^]^ To address this, deep learning models with gating mechanisms, such as long short‐term memory (LSTM) and gated recurrent units (GRU), were introduced. These models provide more accurate SOC predictions. In LSTM, long‐term dependencies are managed by input, forget, and output gates that control the memory flow. GRU, on the other hand, uses a reset and update gate system, resolving long‐term dependency issues but at a lower computational cost compared to LSTM. As a result, data‐driven deep learning methods are gaining significant attention as promising solutions for SOC estimation.^[^ ^192^ ^]^

### 5.2 EKF Method

The EKF method, another popular approach, estimates SOC by calculating the OCV through a battery model. It is well‐regarded for its robustness against noise and ability to provide real‐time estimations, making it commonly used in conjunction with the Coulomb counting method. However, the accuracy of the EKF method relies heavily on the battery model, which must accurately simulate the battery's electrochemical processes and adapt to changes in battery parameters based on operating conditions.

Battery models commonly include the electrochemical (EM) and electrical circuits (ECM). The EM is developed by interpreting the electrochemical processes within a battery cell. While it provides high accuracy in estimating the battery's state, it is challenging to implement. However, recent advancements have led to the development of high‐performance electrochemical model‐based algorithms, such as those based on ensemble Kalman filters.^[^ ^193^, ^194^ ^]^ The ECM represents the battery's behavior using electrical parameters like resistance and capacitance derived from the battery's voltage and current waveforms during charging and discharging. This model offers a less accurate representation than the EM but is more popular due to its simplicity and ease of implementation. In particular, the EKF often relies on a straightforward electrical model using a first‐ or second‐order resistance–capacitance (RC) network, with battery parameters expressed as fixed values, regardless of the SOC range.^[^ ^195^, ^196^, ^197^ ^]^

The EKF incorporates battery parameters into the SOC estimation algorithm through a state‐space equation. As a result, the accuracy of SOC estimation depends on the precision of the battery parameter values.^[^ ^198^ ^]^ Typically, each parameter in the EKF is treated as a constant value. However, these parameters can fluctuate based on factors like ambient temperature, C‐rate, SOC, and charge/discharge cycles.^[^ ^199^, ^200^, ^201^ ^]^ Consequently, using a fixed value for battery parameters in the EKF without accounting for their variability leads to SOC estimation errors. To address this issue, research has been conducted on modifying the EKF to account for changes in battery parameters.^[^ ^186^, ^202^, ^203^ ^]^

In ref. [201], a temperature compensation model was introduced, integrating temperature‐dependent battery parameter variations into the Kalman filter. This model represents the diffusion resistance (*R*_d_) and diffusion capacitance (*C*_d_) as quadratic functions of the SOC, with coefficients that vary according to both temperature and SOC, based on data obtained from offline experiments. As a result, this approach enables more precise SOC estimation than conventional methods that rely on fixed parameter values since the battery parameters are adjusted in real‐time based on temperature and SOC fluctuations.

Other research^[^ ^202^, ^203^ ^]^ explored the use of dual EKFs and dual unscented Kalman filters to enhance the accuracy of battery modelling. By employing two separate filters, these methods account for the dynamic changes in battery parameters over time, allowing for real‐time updates to the battery model and improving the overall SOC estimation accuracy.

Currently, most battery SOC estimation methods are designed for low‐rate discharge conditions. However, as lithium batteries are increasingly used in specialized applications, certain batteries are specifically engineered for high‐rate pulse discharge scenarios.^[^ ^204^ ^]^ Under these conditions, rapid fluctuations occur in internal chemical reactions, temperature distribution, and external characteristics.^[^ ^205^ ^]^ Additionally, the failure boundaries and degradation patterns of these batteries differ from those of conventional low‐rate batteries, leading to significant variations in battery models. As a result, accurately developing an SOC estimation model becomes highly challenging.^[^ ^206^ ^]^

To address this, Ming et al.^[^ ^207^ ^]^ proposed an enhanced LSTM‐RNN model to estimate SOC for ternary lithium batteries operating under high‐rate pulse conditions. Similarly, Jiao et al.^[^ ^208^ ^]^ explored a gated recurrent unit (GRU)‐RNN–based momentum gradient approach, examining how factors such as momentum terms, noise variance, training epochs, and the number of hidden neurons affect training speed and SOC estimation accuracy. Despite these efforts, research on SOC estimation specifically for high‐rate pulse discharge conditions remains limited, and no well‐established methods currently exist to effectively tackle these challenges. Therefore, further investigation in this field holds significant theoretical and practical importance.

Lin et al.^[^ ^209^ ^]^ introduced a method for constructing a fusion model that merges a backpropagation neural network with an unscented Kalman filter (KF). This approach incorporates AdaBoost and recursive least squares to enhance both the accuracy and generalization of SOC estimation in complex operating environments. Similarly, Chen et al.^[^ ^210^ ^]^ developed a hybrid model based on a long short‐term memory recurrent neural network (LSTM‐RNN), featuring extended inputs and constrained outputs, to optimize SOC estimation for electric vehicles (EVs).

### 5.3 Digital Twin Technology

In the field of battery technology, digital twin technology is widely utilized to create a framework that enables seamless data exchange between physical batteries and software systems. This approach enhances the precision of state tracking and improves the reliability of battery management. Yang et al.^[^ ^211^ ^]^ introduced a digital twin framework for lithium‐ion batteries, focusing on predicting their remaining useful life. Their framework incorporates various modules, including data management, model simulation and evolution, and visualization. By developing capacity degradation models, they analyzed the randomness of battery degradation and the inconsistencies in the lifespan of multiple batteries.

The previously mentioned framework primarily emphasizes data filtering, processing, and transmission but does not enhance the underlying models, which are essential for battery management and state estimation. Digital twin technology has the potential to support model evolution—often referred to as a digital twin model—enabling it to continuously approximate the physical battery and improve model accuracy. However, current applications of digital twin technology in battery modeling remain limited, mostly relying on empirical approaches such as data‐driven models^[^ ^212^ ^]^ and equivalent circuit models.^[^ ^213^ ^]^

Qu et al.^[^ ^214^ ^]^ integrated the digital twin concept into lithium‐ion battery performance degradation estimation and proposed a capacity performance degradation evaluation model under dynamic operating conditions. Experimental results indicated that the maximum mean absolute errors for voltage under a 1C constant current condition remained below 0.06 V. Similarly, Yi et al.^[^ ^215^ ^]^ developed a digital twin model based on a lumped thermal equivalent circuit model, incorporating LSTM neural networks for real‐time temperature prediction and battery degradation analysis. Their experiments showed that the maximum temperature errors under 1C constant current and US06 dynamic discharge conditions were 0.31 and 0.85 °C, respectively.

## 6 Summary and Recommendations

EVs are a promising solution to address the global energy crisis and climate change. At present, LiBs serve as a dependable energy storage source for EVs. However, as the demand to reduce charging times and extend driving ranges increases, the heat generated within battery packs during fast charging becomes a significant issue, impacting safety and efficiency. Therefore, an advanced BTMS is crucial for ensuring the safe and efficient use of LIBs in fast‐charging applications. An overview of recent studies on cutting‐edge BTMS technology for EV fast charging carried out throughout the previous 5 years is given in this article. It highlights several cooling methods, like direct and indirect cooling with cooling plates and hybrid cooling systems that combine liquid cooling and PCMs. The following is a summary of the main findings and suggestions from this review:

1. Indirect cooling systems in advanced BTMS face several challenges, such as complex designs, the risk of liquid leakage, potential corrosion, high energy consumption, added weight, and increased maintenance costs. The high thermal resistance between the cooling components and the battery surface further diminishes heat transfer efficiency and overall cooling performance. Therefore, careful evaluation and detailed research are necessary for their use in fast‐charging, high‐capacity battery packs.
2. ICS, also known as direct liquid cooling, is becoming more and more popular as an innovative battery thermal management technique. This method improves heat dissipation by lowering thermal resistance and greatly increasing the heat transfer coefficient when the liquid directly touches the battery cell surface. Compared to single‐phase and indirect approaches, two‐phase cooling using low‐boiling‐point fluids has demonstrated greater efficiency. However, extensive research is needed to understand the interaction of various performance and operational parameters to ensure the feasibility of large‐scale applications, which will guide the design of next‐generation ICSs.
3. PCM cooling, which uses the high latent heat of phase change materials during solid–liquid transitions, offers excellent heat dissipation and temperature uniformity by absorbing the heat generated during fast charging. As a passive cooling method, it lowers energy consumption and saves space. However, PCMs may not meet the heat dissipation requirements of fast‐charging battery packs because of their limited *kp*. As a result, hybrid cooling systems that include PCM and liquid cooling are showing promise as an advanced thermal management solution.
4. Choosing an advanced cooling strategy should consider large‐scale production and operation in harsh environments. Coolant leakage poses a significant risk to system performance in direct liquid cooling. Additionally, the weight of the coolant can make the system heavier, potentially reducing the vehicle's range. Using viscous or dense coolants may also increase pumping power requirements. Therefore, factors such as weight, leakage risks, and pumping power must be evaluated for commercial‐scale production. In extreme environments, cooling systems may experience faster wear due to high temperatures, humidity, and corrosion, necessitating careful material selection and insulation, which adds to the cost.
5. Several suggestions are offered to manufacturers, legislators, and researchers to integrate sophisticated battery heat management in EVs successfully. Choosing environmentally friendly coolants with better heat transfer qualities, adjusting flow distribution according to battery designs, and considering operational factors like flow rate and ambient temperatures are all ways to enhance ICS on a commercial scale. The development of real‐world driving tests and thermal runaway models is necessary to guarantee safe operation in harsh environments. Financial rewards like tax exemptions or subsidies may promote using energy‐saving cooling devices.

This review provides a comprehensive overview of methods to enhance the *kp* of PCMs. However, several technological challenges remain particularly issues like supercooling, which significantly affects the thermal management and stability of PCMs. These problems require further investigation and improvement. Furthermore, despite their effectiveness, hydrous salts are highly corrosive and can harm other materials, which could reduce the longevity of structures and battery packs. Additionally, the cost of PCMs continues to be somewhat high due to the mismatch between supply and demand. Notwithstanding these obstacles, PCMs have a sizable market in the energy management and construction industries, and future growth in their application is anticipated. Therefore, one of the most critical issues that needs to be solved is lowering the cost of PCM manufacture.

## Conflict of Interest

The authors declare no conflict of interest.

## Author Contributions

M.A.R.: Writing—Conceptualization, Data curation, Investigation, Methodology, Original draft; Project administration. G.M.V.R.: Original draft, Validation, Supervision. R.C., S.H., and S.M.M.H.: Writing—review and editing, Software, Methodology. P.P. and L.H.D.: Supervision, Writing—review and editing, Formal analysis, Methodology.
